# Failure to repair damaged NAD(P)H blocks de novo serine synthesis in human cells

**DOI:** 10.1186/s11658-024-00681-8

**Published:** 2025-01-09

**Authors:** Adhish S. Walvekar, Marc Warmoes, Dean Cheung, Tim Sikora, Najmesadat Seyedkatouli, Gemma Gomez-Giro, Sebastian Perrone, Lisa Dengler, François Unger, Bruno F. R. Santos, Floriane Gavotto, Xiangyi Dong, Julia Becker-Kettern, Yong-Jun Kwon, Christian Jäger, Jens C. Schwamborn, Nicole J. Van Bergen, John Christodoulou, Carole L. Linster

**Affiliations:** 1 Enzymology and Metabolism Group, Luxembourg Centre for Systems Biomedicine, University of Luxembourg, L-4367 Belvaux, Luxembourg; 2 Metabolomics Platform, Luxembourg Centre for Systems Biomedicine, University of Luxembourg, L-4367 Belvaux, Luxembourg; 3 Brain and Mitochondrial Research Group, Murdoch Children’s Research Institute, Royal Children’s Hospital, Melbourne, VIC 3002 Australia; 4 Developmental and Cellular Biology Group, Luxembourg Centre for Systems Biomedicine, University of Luxembourg, L-4367 Belvaux, Luxembourg; 5 Disease Modeling and Screening Platform, Luxembourg Centre for Systems Biomedicine, University of Luxembourg, L-4367, Belvaux and Luxembourg Institute of Health, L-1445, Strassen, Luxembourg; 6 Department of Paediatrics, University of Melbourne, Melbourne, VIC 3002 Australia; 7 Victorian Clinical Genetics Services, Royal Children’s Hospital, Melbourne, VIC 3002 Australia

**Keywords:** Metabolite damage and repair, Inborn errors of metabolism, NAD(P)H hydration, NAXD, Serine biosynthesis, 3-Phosphoglycerate dehydrogenase, Inosine, Nicotinamide riboside

## Abstract

**Background:**

Metabolism is error prone. For instance, the reduced forms of the central metabolic cofactors nicotinamide adenine dinucleotide (NADH) and nicotinamide adenine dinucleotide phosphate (NADPH), can be converted into redox-inactive products, NADHX and NADPHX, through enzymatically catalyzed or spontaneous hydration. The metabolite repair enzymes NAXD and NAXE convert these damaged compounds back to the functional NAD(P)H cofactors. Pathogenic loss-of-function variants in *NAXE* and *NAXD* lead to development of the neurometabolic disorders progressive, early-onset encephalopathy with brain edema and/or leukoencephalopathy (PEBEL)1 and PEBEL2, respectively.

**Methods:**

To gain insights into the molecular disease mechanisms, we investigated the metabolic impact of NAXD deficiency in human cell models. Control and NAXD-deficient cells were cultivated under different conditions, followed by cell viability and mitochondrial function assays as well as metabolomic analyses without or with stable isotope labeling. Enzymatic assays with purified recombinant proteins were performed to confirm molecular mechanisms suggested by the cell culture experiments.

**Results:**

HAP1 NAXD knockout (NAXDko) cells showed growth impairment specifically in a basal medium containing galactose instead of glucose. Surprisingly, the galactose-grown NAXDko cells displayed only subtle signs of mitochondrial impairment, whereas metabolomic analyses revealed a strong inhibition of the cytosolic, de novo serine synthesis pathway in those cells as well as in NAXD patient-derived fibroblasts. We identified inhibition of 3-phosphoglycerate dehydrogenase as the root cause for this metabolic perturbation. The NAD precursor nicotinamide riboside (NR) and inosine exerted beneficial effects on HAP1 cell viability under galactose stress, with more pronounced effects in NAXDko cells. Metabolomic profiling in supplemented cells indicated that NR and inosine act via different mechanisms that at least partially involve the serine synthesis pathway.

**Conclusions:**

Taken together, our study identifies a metabolic vulnerability in NAXD-deficient cells that can be targeted by small molecules such as NR or inosine, opening perspectives in the search for mechanism-based therapeutic interventions in PEBEL disorders.

**Graphical Abstract:**

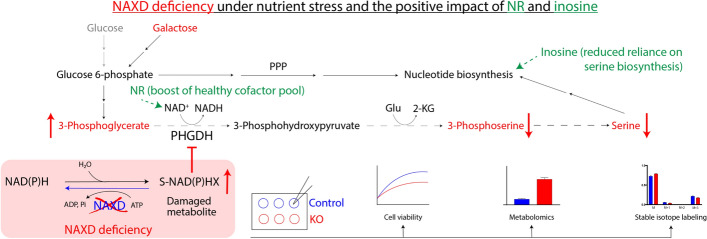

**Supplementary Information:**

The online version contains supplementary material available at 10.1186/s11658-024-00681-8.

## Background

Metabolites are susceptible to damage, owing to either their inherent chemical instability or the promiscuous nature of metabolic enzymes. Damaged metabolites can be either useless or toxic, in which case their concentration needs to be maintained at levels that are low enough to prevent interference with important cellular processes. Cells have developed robust mechanisms to either prevent the formation (preemption) or repair/eliminate damaged metabolites by a set of dedicated enzymes designated “metabolite repair enzymes” [1, 2]. Failure of these repair mechanisms leads to damage accumulation and can have severe consequences on cellular and organismal health. L-2-hydroxyglutaric aciduria was the first inherited metabolic disorder to be identified as a metabolite repair disorder [3], as the deficient enzyme (L-2-hydroxyglutarate dehydrogenase) normally functions to eliminate a promiscuous side product of malate dehydrogenase. Since this original discovery, four additional disorders of metabolite repair have been uncovered [4]. More “damaged” or noncanonical metabolites and corresponding enzymatic repair systems continue to be discovered [2] and investigated in the context of unsolved rare metabolic diseases.

**Fig. 1:**
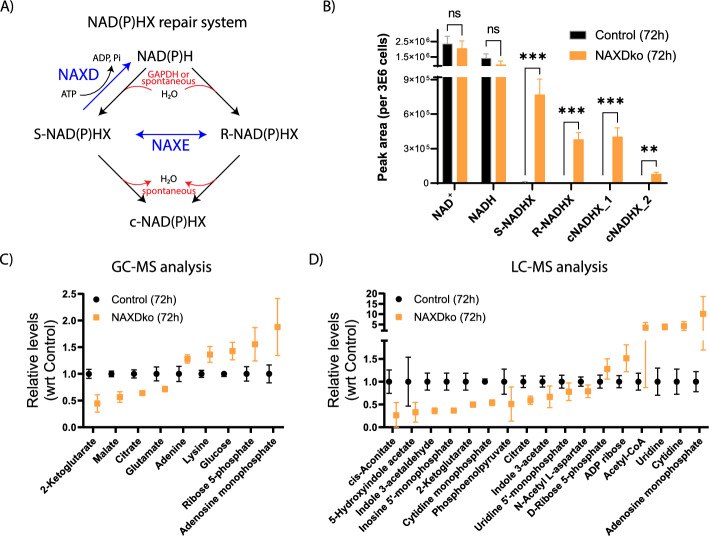
Metabolic profiling of a HAP1 cell model of NAXD deficiency in standard (Iscove’s modified Dulbecco’s medium, IMDM) medium. **A** Schematic presentation of the NAD(P)HX repair system. Hydration damage (enzymatic or nonenzymatic) of NAD(P)H generates *S*- or *R*-NAD(P)HX forms that can interconvert and further react to form cyclic forms designated collectively as cNAD(P)HX. The repair enzymes (NAXD and NAXE) are shown in blue. The NAXE epimerase accelerates interconversion between the *S*- and *R*-NAD(P)HX forms, whereas the NAXD dehydratase stereospecifically acts on *S*-NAD(P)HX in an ATP-dependent manner. **B** HAP1 NAXDko cells accumulate damaged forms of NADH. Control and NAXDko cells were grown in IMDM for 72 h or 96 h (Supplementary Fig. S1A), and NAD(H)(X) metabolites were measured by LC–MS. All values were normalized to an internal standard and cell counts from replicate plates, and are means ± SDs of three replicate wells. Statistical significance was calculated using an equal variance, unpaired Student’s *t*-test. **p < 0.01; ***p < 0.001; *ns*, not significant. **C**, **D** Metabolite extracts from HAP1 control and NAXDko cells grown for 72 h and 96 h in IMDM were analyzed using untargeted GC–MS (C) and LC–MS (D) methods (72 h data is shown here, whereas 96 h data is shown in Supplementary Fig. S1B, C). For both the GC–MS and LC–MS data, metabolites with significant changes between the control and NAXDko cells at both time points are presented. For the LC–MS data plotted in Fig.1D and Supplementary Fig. S1C, the presence of metabolites in significantly enriched metabolite sets was used as an additional selection filter. GC–MS data were first normalized using the internal standard [U-^13^C]-ribitol. LC–MS data were normalized using the summed MS1 intensities of all metabolites within each sample. For both GC–MS and LC–MS data, all values presented are relative to controls and are means ± SDs of six biological replicates. Pathway analysis for the differential metabolites detected by LC–MS is presented in Supplementary Fig. S2. The detailed list of metabolites is compiled in Additional file 1. NAXD, NAD(P)HX dehydratase; NAXE, NAD(P)HX epimerase; GAPDH, glyceraldehyde 3-phosphate dehydrogenase; Acetyl-CoA, acetyl coenzyme A; ADP, adenosine diphosphate

The central metabolic cofactors NADH and NADPH are prone to hydration damage, forming NAD(P)HX derivatives that are hydroxylated at the nicotinamide ring and can be under the *R*- or *S*-epimeric forms, or react further to generate cyclic forms [5] (Fig. 1A). All these derivatives are redox inactive and can inhibit dehydrogenases owing to their structural similarity to the normal nicotinamide cofactors [6–8]. A pair of metabolic enzymes, encoded by the *NAXD* and *NAXE* genes, convert the hydrated derivatives back to the normal NAD(P)H cofactors [9]. Both enzymes are conserved in all forms of life, suggesting the universal occurrence of the damage, as well as the need to correct it. NAXD is essential for the repair as it stereospecifically converts *S*-NAD(P)HX to NAD(P)H in an adenosine triphosphate (ATP)-dependent manner. NAXE catalyzes the interconversion between the *R*- and *S*-NAD(P)HX forms, essentially optimizing the repair system. Both the *NAXD* and *NAXE* genes encode several isoforms that are targeted to different subcellular compartments (cytosol, mitochondria, and also endoplasmic reticulum in the case of NAXD) [10], suggesting that maintenance of a healthy pool of NAD(P)H is necessary for all these compartments.

More recently, a causal connection between NAXD deficiency and a lethal neurodegenerative disease has been established [11]. Infants with NAXD deficiency are normal at birth, without any apparent developmental defects. The onset of the disease coincides with neurological regression crises induced by febrile and potentially other triggers, rapidly progressing to affect brain and heart function, along with the development of characteristic skin lesions [12, 13]. Since the first reported cases in 2019, several new patients have been identified [14–19]. Patients with NAXE deficiency display a very similar disease course, triggered by febrile illness [20–26]. Apart from its role in NAD(P)HX clearance, NAXE has been reported to moonlight as an apolipoprotein A-I binding protein (hence its original designation as AIBP) [27, 28]; it remains unclear whether and how this additional function contributes to disease development. Recently, *n*-of-1 trials with high-dose administration of the NAD precursor niacin to patients with NAXD and NAXE deficiency have shown promising clinical improvements [13, 18]. However, the early molecular and cellular perturbations determining disease development in NAXD and NAXE deficiency remain unknown, as is the mechanism through which niacin exerts its beneficial effects.

Understanding the metabolic impact of NAXD deficiency in simpler experimental models is thus crucial to gain deeper insights into the pathomechanisms of the human disease. NAXD deficiency has been studied in a budding yeast model [8], and preliminary results have been obtained in patient-derived fibroblasts [11, 19] and HAP1 cells (a near-haploid cell line derived from a chronic myelogenous leukemia patient) [8]. In the NAXD-deficient yeast model [8], NADHX damage increased in stationary phase cultures and during cultivation at higher temperatures. Transcriptomic and metabolomic analyses of NAXD-deficient yeast cells revealed perturbations in serine metabolism as the most striking feature [8]. Further investigation identified blockage of the first step in the serine biosynthesis pathway, due to a potent inhibition of phosphoglycerate dehydrogenase by NADHX as the reason underlying this metabolic perturbation. Preliminary analysis of NAXD-deficient HAP1 cells showed increased glucose consumption and lactate secretion, potentially indicating mitochondrial dysfunction [8]. Accordingly, decreased levels of respiratory chain complex I and IV subunits were detected in fibroblasts from NAXD patients, and those cells showed impaired growth in galactose media as another indicator of mitochondrial dysfunction [11]. Significant mitochondrial proteomic impairments, including reduced levels of respiratory complexes I and IV as well as the mitoribosome, and upregulated mitochondrial apoptotic pathways have also been described in a cohort of patient-derived fibroblasts [19]. However, it is yet to be investigated whether serine biosynthesis is perturbed in human cell models of NAXD deficiency. Of note, yeast 3-phosphoglycerate dehydrogenase (PHGDH) catalyzes 3-phosphoglycerate oxidation via a distinct (transhydrogenase) mechanism compared with human PHGDH [29], making it difficult to predict whether the susceptibility of PHGDH to NADHX inhibition is conserved in humans.

Here, we studied the effects of NAXD deficiency in HAP1 and patient-derived fibroblast cell models using growth assays, various metabolomic analyses, and stable isotope labeling under glucose and galactose conditions. NAXD-deficient cells showed growth inhibition specifically under galactose stress, and untargeted metabolomic analyses in NAXD-deficient HAP1 cells revealed, in an unbiased manner, inhibition of de novo serine synthesis as a main metabolic perturbation. Whole brain organoids derived from NAXDko human induced pluripotent stem cells (iPSCs) also showed high NADHX accumulation and signs of impaired serine biosynthesis. In vitro investigations demonstrated that recombinant human PHGDH is inhibited by NADHX, revealing the most likely cause underlying perturbed serine metabolism in the cell models. Finally, we showed that inosine and the NAD precursor nicotinamide riboside (NR) partially rescue the growth phenotype of NAXD-deficient HAP1 cells under galactose stress, presumably via distinct effects of both compounds on the serine synthesis pathway as suggested by our metabolomic analyses.

## Results

### Absence of NAXD in HAP1 cells alters central metabolic pathways in a standard medium

It has been observed previously that, during short-term cultivations on glucose medium (up to 72 h), the viability of NAXDko cells is similar to that of control cells [8]. Here, we wanted to explore whether, despite this apparently normal growth behavior, differences could be detected at the metabolome level in NAXD-deficient cells in standard cultivation medium (IMDM). For this purpose, HAP1 control and NAXDko cells were grown in this medium for 72 h or 96 h followed by metabolite extraction and analysis using different techniques. NAD(H)(X) compounds were measured using a previously established method based on liquid chromatography–mass spectrometry (LC–MS) [8]. These measurements showed a clear accumulation of the different forms of NADHX in the NAXDko cells at both time points, with no significant changes in the levels of the normal NAD(H) cofactors (Fig. 1B and Supplementary Fig. S1). To probe metabolic perturbations more broadly, we also analyzed the samples using untargeted GC–MS and liquid chromatography with tandem mass spectrometry (LC–MS/MS) methods. Both GC– and LC–MS analyses were performed in triplicate (extracts from replicate cultivation wells) for the 72 h and 96 h time points, and experiments were performed twice on two different days. Both independent datasets were combined and analyzed by scaling the values from control cells to 1. Only metabolites that had significantly different levels at both time points were considered for further data analysis and interpretation. In that sense, nine metabolites measured by GC–MS and annotated based on an in-house mass spectral library (confidence level 1) showed significant differences between control and NAXDko cells (Fig. 1C and Supplementary Fig. S1B). Specifically, at both time points, three tricarboxylic acid (TCA) cycle intermediates (citrate, 2-ketoglutarate, and malate) and glutamate were decreased in the NAXDko cells, whereas adenine, lysine, glucose, ribose-5-phosphate, and adenosine monophosphate (AMP) were increased. Based on the LC–MS/MS analysis, a total of 177 metabolites, annotated on the basis of public mass spectral libraries (i.e., with low confidence), were found to have significantly different levels at both the 72 h and 96 h time points. Among these, 103 metabolites were significantly increased and 57 metabolites were significantly decreased in NAXDko cells, and 17 metabolites showed changes in opposite directions in control and NAXDko cells for the two time points. For the differential metabolites found by LC–MS/MS analysis, we performed a metabolite enrichment analysis using the Kyoto Encyclopedia of Genes and Genomes (KEGG) database (Supplementary Fig. S2). A total of seven metabolite sets were significantly enriched with a total of 17 differential metabolites represented in these seven sets (Fig. 1D and Supplementary Figs. S1C and S2A, B). The most significantly enriched metabolite set was the TCA cycle, in good agreement with the results from the GC–MS analysis. Both pyrimidine and purine metabolite sets were also significantly enriched. Of note here, several nucleotide monophosphates (IMP, UMP, and CMP) were found to be decreased in the NAXDko cells, while two of the corresponding nucleoside counterparts (cytidine and uridine) accumulated in the NAXDko cells. The only nucleotide monophosphate that showed accumulation in the NAXDko cells, based on the GC–MS and LC–MS/MS analyses, was AMP. The third most significantly enriched metabolite set was tryptophan metabolism, with decreased levels of the tryptophan catabolites 5-hydroxyindoleacetate, indole 3-acetaldehyde, and indole 3-acetate in NAXDko cells, potentially due to increased usage of tryptophan for de novo NAD synthesis. Overall, the analyses at both time points (72 h and 96 h) pointed mainly toward altered mitochondrial and nucleotide metabolism in NAXDko cells, but further work is needed to validate those findings by targeted analyses and understand how these changes are linked to NAXD deficiency.

## NAXDko cells show impaired growth on galactose, with subtle alterations of mitochondrial function

**Fig. 2:**
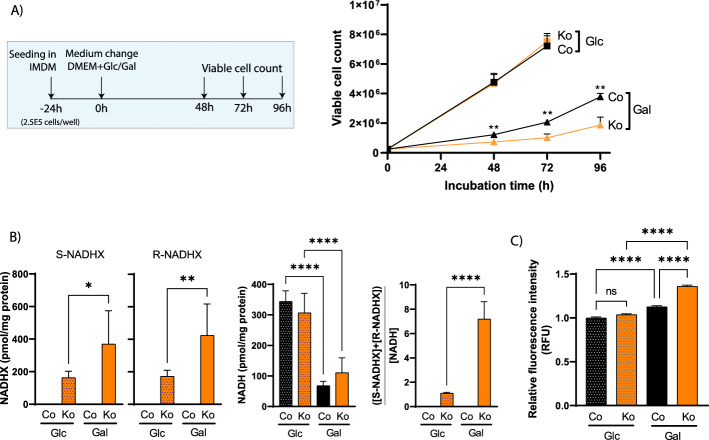
NAXD-deficient HAP1 cells show more pronounced phenotypes under galactose culture conditions. **A** The HAP1 NAXDko cells show impaired growth specifically in galactose medium. The experimental design is shown in the inset. HAP1 control and NAXDko cells were seeded in IMDM and allowed to grow for 24 h, and then the medium was changed to a basal medium with either glucose (25 mM) or galactose (25 mM). **B** Control and NAXDko cells were grown in glucose or galactose as indicated in panel **A**, and *S*-NADHX, *R*-NADHX, and NADH levels were measured after 72 h by HPLC–UV (NADPH and NAD^+^ measurements are shown in Supplementary Fig. S3). The NADHX burden (calculated as the sum of *S*- and *R*-NADHX levels divided by the NADH level) was increased in NAXDko cells grown in galactose compared with the same cells grown in glucose. **C** NAXDko cells grown in galactose show increased mitochondrial superoxide levels. HAP1 control and NAXDko cells were grown in glucose or galactose as indicated in panel **A**, cells were stained with MitoSOX Red after 72 h, and images were captured at an excitation wavelength of 488 nm. Fluorescence intensity was measured as relative fluorescence units (RFUs) and normalized to control cells grown in glucose. All values are means ± SDs of 3–6 biological replicates, and statistical significance was calculated using an equal variance, unpaired Student’s *t*-test. *p < 0.05; **p < 0.01, ****p < 0.0001; ns, not significant. For all panels, Co, Control; Ko, NAXDko; Glc, glucose medium; Gal, galactose medium

As our metabolomic analyses in standard IMDM medium indicated perturbed mitochondrial metabolism, and given the (among others) mitochondrial targeting of the NAXD enzyme [10], we next focused on studying NAXDko cells cultivated in galactose medium, a condition known to render cells more dependent on metabolically functional mitochondria [30]. A decreased live cell count was obtained at all time points for the NAXDko cell line compared with the control line in galactose medium, whereas both cell lines showed a similar viability in glucose medium (Fig. 2A). HPLC–UV measurements revealed an approximately twofold increase in *S*- and *R*-NADHX levels for NAXDko cells grown in galactose medium compared with the ones grown in glucose medium (Fig. 2B). Although the levels of NAD^+^ and NADPH remained unchanged upon *NAXD* deletion (Supplementary Fig. S3), we found overall decreased NADH levels for control and NAXDko cells when glucose was replaced by galactose in the cultivation medium (Fig. 2B). Consequently, in the galactose condition, NAXDko cells carried an increased “NADHX burden,” calculated as the ratio of damaged versus intact NADH (sum of *S*-NADHX + *R*-NADHX/NADH) (Fig. 2B). Measurements of cyclic forms of NADHX in the HAP1 cell extracts using HPLC–UV were not reliable owing to co-elution with interfering compounds and thus were not included in this calculation. It can be noted that our observation of unchanged NAD^+^ levels and decreased NADH levels in our cells (control *and* NAXDko) when cultivated in galactose versus glucose medium agrees with increased NAD^+^/NADH ratios reported previously for HAP1 cells cultivated in the presence of galactose [31]. Owing to the increased NADHX burden, some NAD-dependent enzymes may be more strongly (competitively) inhibited by NADHX in the galactose medium, which in turn may contribute to the decreased cell viability of the NAXDko cells under this specific condition.

Given the sensitivity of NAXDko cells to galactose stress, we performed morphometric analyses using high-content imaging to test general features and functionality of mitochondria (Supplementary Fig. S4). These analyses revealed no significant differences between NAXDko and control cells in mitochondrial count, total mitochondrial area (both normalized to nuclear count), or membrane potential on glucose or galactose media. The only parameter that was significantly different between control and NAXDko cells, and only under the galactose condition (irrespective of any additional supplementations or treatments), was the nuclear count, which confirmed decreased viability of NAXDko cells specifically in this condition (Supplementary Fig. S4). Second, we carried out Seahorse analyses to measure oxygen consumption rate (OCR) in control and NAXDko cells, both in the basal state and after the successive addition of oligomycin, FCCP, or antimycin A/rotenone (Supplementary Fig. S5). Owing to poor adhesion of the cells to the Seahorse plate in galactose-supplemented media, we could not perform the Seahorse assays conclusively in this condition. Under glucose supplementation, no difference in OCR could be detected between control and NAXDko cells in IMDM, but we consistently found a significantly decreased OCR in the NAXDko cells in Dulbecco’s modified Eagle’s medium (DMEM) medium, where overall substantially higher OCR values were measured as for IMDM-cultivated cells. Third, we performed a tracer experiment using ^13^C_5_-glutamine to test the reductive carboxylation potential of control and NAXDko cells grown in glucose or galactose medium. Mitochondrially impaired cells may show increased reductive carboxylation through the reverse TCA cycle, indicated by increased formation of M + 5 citrate, M + 3 malate, and M + 3 fumarate [32]. The NAXDko cells showed a small increase in reductive carboxylation of glutamine in glucose medium, but this tendency was even inverted in galactose medium (Supplementary Fig. S6). Lastly, to test whether mitochondrial oxidative stress was altered in NAXDko cells, we performed mitochondrial superoxide staining with the MitoSOX dye (Fig. 2C). This analysis revealed a significant and selective increase of mitochondrial oxidative stress in galactose-grown NAXDko cells compared with control cells. Overall, these observations indicated that, while mitochondrial health may be subtly altered in NAXDko cells, these alterations are unlikely to fully account for the growth inhibition observed for those cells under galactose stress.

## Metabolomic profiling of NAXDko cells under galactose stress reveals perturbed de novo serine synthesis

As a number of the mitochondrial parameters measured here were not significantly altered, we speculated that the growth inhibition of galactose-grown NAXDko cells could be due to perturbed cytosolic pathway(s), such as pentose phosphate pathway (PPP), nucleotide metabolism, or glycolysis. To reach higher coverage of intermediates in these pathways, we analyzed metabolites extracted from galactose-grown cells by ion chromatography coupled to mass spectrometry (ICMS), which is well suited for resolution and quantification of highly polar metabolites [33], and also by hydrophilic interaction liquid chromatography-mass spectrometry (HILIC-MS). As the NAXDko cells showed growth inhibition in galactose medium, a higher number of NAXDko cells was seeded compared with control cells to reach similar cell counts at the time of extraction for both cell lines (Supplementary Fig. S7A).

**Fig. 3:**
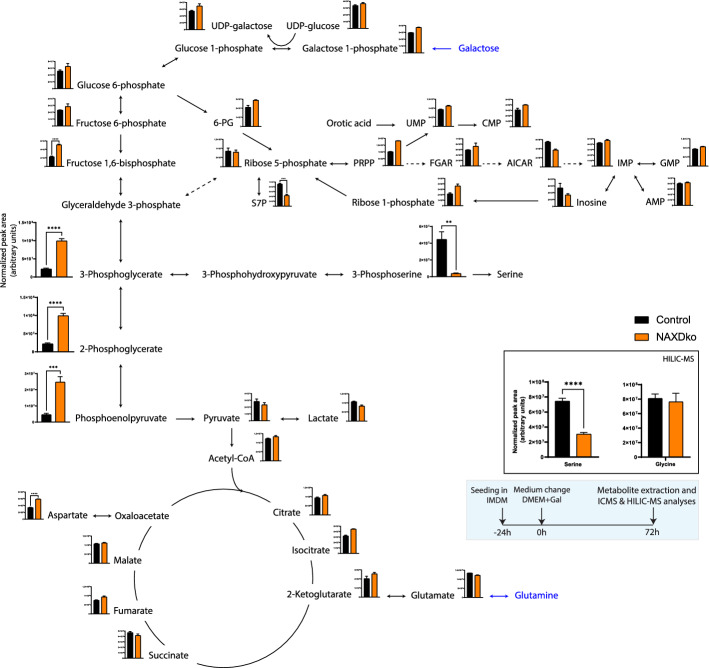
NAXD-deficient HAP1 cells grown in galactose medium show perturbations in the serine biosynthesis pathway. The experimental scheme is shown in the blue inset. Control and NAXDko cells were seeded in IMDM and shifted to galactose-containing medium for 72 h before metabolite extraction and analysis by ICMS and HILIC-MS methods. The metabolite levels (peak areas) were normalized to summed MS1 peak intensities and mapped manually on the indicated central metabolic pathways. The HILIC analysis was performed to determine serine and glycine levels (shown in the other inset). The nutrient sources are marked with blue font. All values are means ± SDs of three biological replicates. Statistical significance was calculated using an equal variance, unpaired Student’s *t*-test; nonsignificant changes (*p*-value > 0.05) are not annotated with a symbol. **p < 0.01, ***p < 0.001; ****p < 0.0001. 6-PG, 6-phosphogluconate; S7P, sedoheptulose 7-phosphate; PRPP, phosphoribosyl pyrophosphate; FGAR, phosphoribosyl-*N*-formylglycinamide; AICAR, 5-aminoimidazole-4-carboxamide ribonucleotide; AMP, adenosine monophosphate; GMP, guanosine monophosphate; IMP, inosine monophosphate; CMP, cytidine monophosphate; UMP, uridine monophosphate; UDP, uridine diphosphate; Acetyl-CoA, acetyl coenzyme A; Gal, galactose; ICMS, ion-exchange mass spectrometry; HILIC-MS, hydrophilic interaction liquid chromatography- mass spectrometry

Metabolite levels measured by ICMS in extracts of cells cultivated in DMEM containing either glucose or galactose were mapped on the central metabolic pathways (Supplementary Fig. S7B and Fig. 3). In contrast to our preliminary observations under standard (IMDM) culture conditions (Fig. 1C, D), we did not observe any significant differences in the levels of TCA, PPP, or nucleotide metabolism intermediates for cells grown in galactose (Fig. 3; differences were also attenuated for those pathways in the DMEM glucose (Supplementary Fig. S7B) versus the IMDM condition (Fig. 1C, D)). Interestingly, the NAXDko cells cultured in galactose showed significant accumulation of the lower glycolytic triose phosphate intermediates with a concomitant depletion of 3-phosphoserine, a metabolite formed from 3-phosphoglycerate via the de novo serine synthesis pathway (Fig. 3). The NAXDko cells grown in a basal medium with glucose showed neither 3-phosphoglycerate accumulation nor 3-phosphoserine depletion (Supplementary Fig. S7B), suggesting that inhibition of the serine synthesis pathway is specific to or at least significantly more pronounced under galactose stress. Although the cultivation medium contained exogenous serine and glycine, we also measured the intracellular levels of those end-products of the serine synthesis pathway by HILIC-MS (Fig. 3, inset). The serine levels were significantly decreased in NAXDko cells compared with control cells, indicating the active contribution of the serine biosynthetic pathway to the intracellular pool of serine in galactose-grown cells. The levels of glycine, which can be formed from serine, were not changed in the NAXDko cells, possibly owing to its compensatory synthesis via other routes (e.g., from threonine, choline, or glyoxylate [34]) or to increased uptake of extracellular glycine. NAXEko cells, which accumulate approximately 100-fold less *S*-NADHX and tenfold less *R*-NADHX in comparison with NAXDko cells [8], did not show any signs of inhibition of the serine synthesis pathway when grown in a basal medium with galactose (Supplementary Fig. S7C). Taken together, these observations clearly indicate that intracellular NADHX accumulation can lead to inhibition of de novo serine synthesis in human cells.

## In vivo and in vitro demonstration of the inhibition of human PHGDH by NADHX

**Fig. 4:**
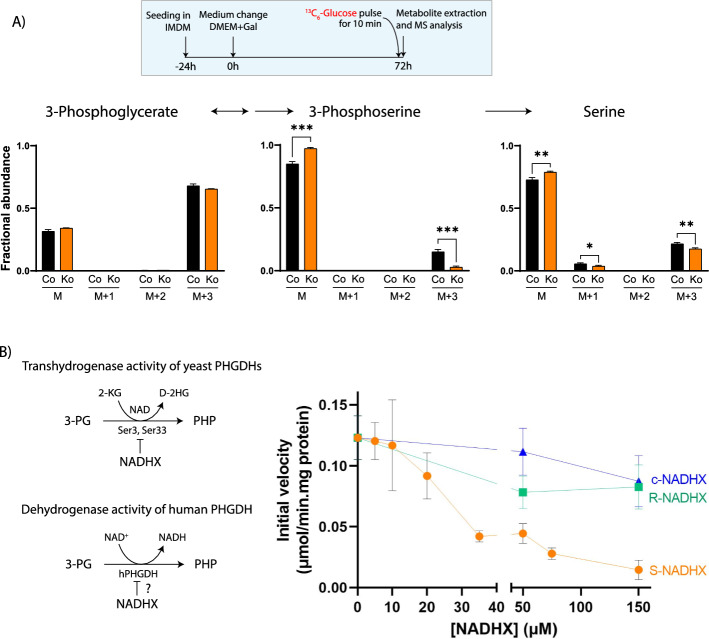
NAXD-deficient HAP1 cells grown in galactose medium show inhibition at the PHGDH step of serine biosynthesis. **A** Tracer analysis with ^13^C_6_-glucose demonstrates inhibition of the serine pathway in living NAXDko cells. The experimental scheme is shown in the inset. Control and NAXDko cells grown in galactose were pulsed with ^13^C_6_-glucose for 10 min or 30 min (Supplementary Fig. S9A) followed by metabolite extraction and LCMS (ICMS and HILIC-MS) analysis. Fractional distribution of the label is shown for 3-phosphoglycerate, 3-phosphoserine, and serine. The labeling data for glycine, 6-phosphogluconate, and lactate are presented in Supplementary Fig. S9B. All values are means ± SDs of three biological replicates. Statistical significance was calculated using an equal variance, unpaired Student’s *t*-test. *p < 0.05; **p < 0.01; ***p < 0.001. **B** The dehydrogenase activity of recombinant human PHGDH is inhibited by NADHX. The difference in reaction mechanism catalyzed by the yeast (Ser3 and Ser33) and human (hPHGDH) PHGDHs is shown on the left. The assay was performed with purified, recombinant PHGDH (purification gel is shown in Supplementary Fig. S10A) at a substrate (3-PG) concentration of 40 µM and in the absence and presence of the indicated concentrations of purified *S*-, *R*-, or c-NADHX forms. All values are means ± SDs of at least three independent measurements. Co, Control; Ko, NAXDKo; Gal, galactose; MS, mass spectrometry; 3-PG, 3-phosphoglycerate; PHP, 3-phoshohydroxypyruvate; 2-KG, 2-ketoglutarate; D-2HG, D-2-hydroxyglutarate; PHGDH, 3-phosphoglycerate dehydrogenase

To test inhibition of de novo serine synthesis in vivo more directly, we performed stable isotope labeling experiments under galactose culture conditions. Tracing with ^13^C_5_-glutamine for 24 h (Supplementary Fig. S8) or 72 h (not shown) showed reduced label incorporation into 3-phosphoserine in NAXDko cells compared with the control cells. However, this experimental design allowed for only limited label incorporation into 3-phosphoglycerate and relied on a medium exchange for fresh tracer medium (that also contained 10% fetal bovine serum), potentially further diluting the label with unlabeled glutamine. We also tried to use ^13^C_6_-galactose as a tracer, but owing to slow growth of both the control and NAXDko cells under this condition, we did not proceed with this tracer experiment (data not shown). To achieve more efficient label incorporation into glycolytic pathway intermediates, we switched to a pulse labeling approach, exposing the cells growing on nonlabeled galactose to ^13^C_6_-glucose for shorter periods (10 min and 30 min) toward the end of the cultivation time (Fig. 4A, inset). This approach yielded good and similar label incorporation into 3-phosphoglycerate in both control and NAXDko cells (Fig. 4A and Supplementary Fig. S9A). A clear decrease in labeling for 3-phosphoserine and serine was observed in NAXDko versus control cells (Fig. 4A and Supplementary Fig. S9A), providing in vivo evidence for inhibition of the serine synthesis pathway in galactose-grown NAXDko cells. The glucose spiking experiment indicated relative differences in label incorporation into some additional metabolites, notably glycine, 6-phosphogluconate, and lactate (Supplementary Fig. S9B). Decreased label incorporation into glycine may be a consequence of the serine biosynthesis pathway inhibition. Reduced label incorporation into 6-phosphogluconate in NAXDko cells could reflect decreased activity of glucose 6-phosphate dehydrogenase, an enzyme previously shown to be inhibited in vitro by NADPHX [7]. Finally, the slightly increased labeling of lactate in the NAXDko cells may result from the moderate mitochondrial impairments described above and in prior work [11, 19] and is in agreement with higher extracellular lactate levels measured previously in NAXDko cells [8].

Accumulation of upstream (3-phosphoglycerate) and depletion of downstream (3-phosphoserine) intermediates of the serine biosynthetic pathway in galactose-grown NAXDko cells suggested a metabolic blockage at the first step of this pathway, catalyzed by the NAD-dependent enzyme 3-phosphoglycerate dehydrogenase (PHGDH). We found no differences in PHGDH protein levels between control and NAXDko cells using western blot analyses, suggesting that the changes in the serine synthesis pathway intermediate levels in the NAXDko cells are not caused by decreased PHGDH expression (Supplementary Fig. S10B). In previous work, we found serine synthesis to be impaired in a yeast model of NAXD deficiency [8] and demonstrated inhibition of the transhydrogenase activity of yeast PHGDH (encoded by the *SER3* and *SER33* genes) by different forms of NADHX to be the mechanism underlying this perturbation [8]. As the human PHGDH (a bona fide dehydrogenase [29]) oxidizes 3-phosphoglycerate via a different catalytic mechanism from the yeast enzyme (transhydrogenase activity relying on 2-ketoglutarate as final electron acceptor, with enzyme-bound NAD as an intermediate acceptor), inhibition of human PHGDH by NADHX could not be directly implied based on the findings with the yeast enzyme (Fig. 4B schematic). We therefore heterologously expressed and purified human PHGDH (Supplementary Fig. S10A) and assayed its dehydrogenase activity in the presence of different concentrations of *S*-, *R*-, or cyclic NADHX (Fig. 4B). At subsaturating substrate (3-phosphoglycerate) concentrations, we found a dose-dependent inhibition of the dehydrogenase activity by *S*-NADHX and, as for the yeast homologous enzyme, we observed less potent inhibition with *R*-NADHX and cyclic NADHX (Fig. 4B). To test whether PHGDH is posttranslationally modified, specifically in galactose medium, in a way that renders it more susceptible to NADHX inhibition, we performed the 3-phosphoglycerate dehydrogenase assay with protein extracts obtained from glucose- or galactose-cultured cells (Supplementary Fig. S10C). The dehydrogenase activity was equally susceptible to *S*-NADHX inhibition in cell extracts derived under both cultivation conditions, indicating that endogenous PHGDH was not modified per se, and that it was more likely the metabolic rewiring induced using galactose instead of glucose that unmasked the serine synthesis phenotype under NAXD deficiency.

Taken together, our in vivo tracer experiments and in vitro enzymatic analyses of human PHGDH demonstrated inhibition of the serine biosynthesis pathway in galactose-grown NAXDko cells at the first committed (PHGDH) step by NADHX, with the most potent effect measured with *S*-NADHX.

## Genetic rescue of NAXD deficiency points toward compartment-specific contributions to the metabolic phenotype

**Fig. 5:**
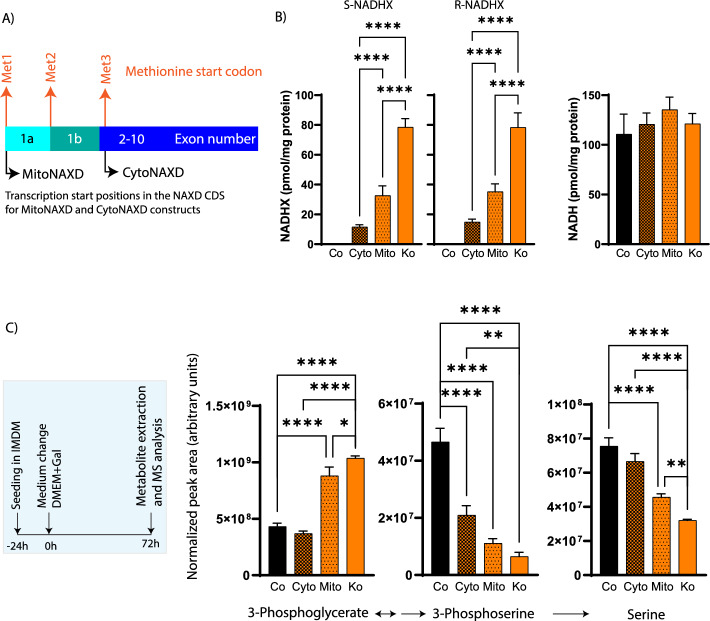
Cytosolic, but not mitochondrial, expression of NAXD in NAXD-deficient HAP1 cells relieves inhibition of serine biosynthesis. **A** Schematic presentation of the human *NAXD* gene showing the transcription start sites of the different NAXD isoforms. The CytoNAXD and MitoNAXD rescue lines were generated by lentiviral transduction of the corresponding constructs into NAXDko cells. Validation of the rescue lines by qPCR analysis is presented in Supplementary Fig. S11A. **B** All cell lines were grown in a standard IMDM medium and *S*-NADHX, *R*-NADHX, and NADH levels were measured by HPLC–UV after 72 h (NADPH and NAD^+^ levels are shown in Supplementary Fig. S11B). **C** Intracellular levels of serine pathway metabolites were measured (by LCMS) after 72 h in galactose medium as shown in the inset. Data were normalized using the summed MS1 intensities of all metabolites within each sample. The levels of other lower glycolytic intermediates are shown in Supplementary Fig. S11C. All values are means ± SDs of six (panel **B**) and three (panel **C**) biological replicates. Statistical significance was calculated using ordinary one-way ANOVA (*p < 0.05; **p < 0.01, ****p < 0.0001). Co, Control_eGFP; Cyto, CytoNAXD rescue line; Mito, MitoNAXD rescue line; Ko, NAXDko_eGFP; Gal, galactose; MS, mass spectrometry

We next wanted to test whether the serine synthesis inhibition in NAXDko cells could be rescued by genetic means. To address this question in a subcellular compartment-specific manner, we generated two rescue lines in the NAXDko background, overexpressing untagged versions of the cytosolic (CytoNAXD) and the mitochondrially targeted (MitoNAXD) isoforms of NAXD, respectively (Fig. 5A). As we lack a NAXD antibody that specifically detects NAXD in cell extracts, we measured the mitochondrial and cytosolic mRNA transcripts using specific primer pairs by quantitative PCR (qPCR) (Supplementary Fig. S11A). We detected increased levels of an amplicon derived from the mitochondrial targeting sequence (MTS) only in the MitoNAXD rescue line whereas increased levels of a “cytosolic” amplicon (derived from a sequence common to both isoforms) were found in both the MitoNAXD and CytoNAXD lines (Supplementary Fig. S11A). In our previous work, we confirmed the predicted subcellular localizations of the different NAXD isoforms (fused to different C-terminal tags) using fluorescence microscopy [10]. As a functional validation step, we measured the levels of normal and damaged NAD(P)H cofactors in these cell lines grown in standard IMDM medium by HPLC–UV (Fig. 5B and Supplementary Fig. 11B). Whereas the levels of NADPH were decreased in NAXDko cells and showed a partial rescue in the CytoNAXD line but not in the MitoNAXD line, the NAD(H) levels remained largely unchanged. We observed markedly reduced levels of *R*- and *S*-NADHX in the rescue lines. The CytoNAXD line showed a near-complete rescue of NADHX levels to control levels (Fig. 5B), while the MitoNAXD line showed a more partial reduction, suggesting that a majority of the “repairable” NADHX pool may be located in the cytosol. We then carried out the ICMS and HILIC-MS analyses in the galactose-grown rescue lines, along with the appropriate control lines. The ICMS analysis showed a near-complete rescue for 3-phosphoglycerate levels in the CytoNAXD line, but only a very partial rescue for this metabolite in the MitoNAXD line (Fig. 5C), indicating that cytosolic expression of NAXD (and thus clearance of cytosolic NADHX) efficiently relieves inhibition at the first step of the serine synthesis pathway. Other lower glycolytic triose phosphate intermediates also showed a similar rescue in levels specifically in the CytoNAXD line (Supplementary Fig. S11C), again suggesting that the major bottleneck is relieved upon cytosolic clearance of NADHX. While we also observed a near-complete rescue for serine levels in the CytoNAXD line, the rescue effect for 3-phosphoserine levels was only partial, for reasons that remain unclear (Fig. 5C). In addition to 3-phosphoserine synthesis, the enzyme 3-phosphoserine aminotransferase (PSAT1) also contributes toward formation of 2-ketoglutarate, which feeds into mitochondrial metabolism [35]. Therefore, we speculate that both cytosolic as well as mitochondrial compartments need to function optimally to fully relieve inhibition of the serine synthesis pathway.

The expression of untagged NAXD isoforms in our rescue lines, as well as the lack of a specific antibody for NAXD detection in cell extracts or intact cells, prevented estimation of NAXD protein expression levels in cytosol versus mitochondria. Assuming, based on our qPCR analyses and previous work [10], that our CytoNAXD and MitoNAXD rescue lines express mostly increased levels of cytosolic and mitochondrial NAXD, respectively, our results suggest, however, a more critical role for cytosolic NADHX clearance in preserving de novo serine synthesis flux.

## Inosine and NR supplementations partially rescue the galactose growth phenotype in NAXDko cells

We also sought to rescue the growth phenotype and/or metabolic blockage at the PHGDH step in galactose-grown NAXDko cells by small-molecule supplementation. Three candidate molecules were tested: serine (end-product of the blocked metabolic pathway), NR (a well-known NAD precursor that directly feeds into NAD biosynthesis [36]), and inosine (which showed consistently lower intracellular levels in NAXDko cells; Fig. 3). Supplementation of the latter had previously been shown to exert beneficial effects on galactosemic fibroblasts [37] as well as on T cells [33], grown under galactose stress, as an alternative and efficient ribose donor. In addition, niacin therapy has shown promising results in alleviating disease symptoms of a few NAXD [14, 18] and NAXE [13, 23] patients. NR supplementation could, in principle, increase the NAD^+^/NADH ratio [38] and relieve the inhibition of NAD-dependent enzymes (such as PHGDH).

**Fig. 6:**
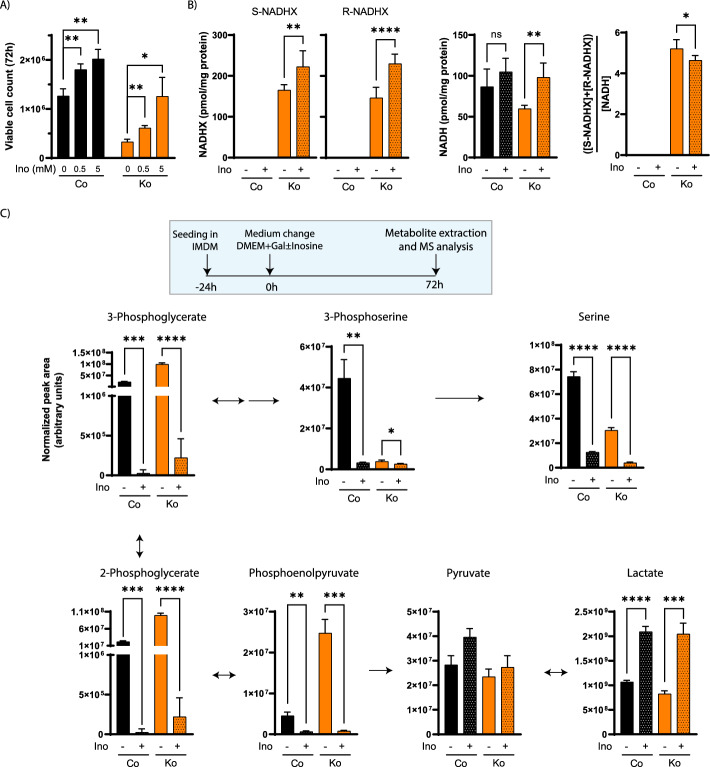
Inosine partially rescues growth of NAXD-deficient HAP1 cells and drastically reduces de novo serine synthesis. **A** Inosine supplementation shows dose-dependent, partial rescue in growth of NAXDko cells. HAP1 control and NAXDko cells were grown in galactose media without or with supplementation of inosine at the indicated concentrations. The viable cell count was measured after 72 h (the count after 96 h and replots of the time course data relative to the control cell viability are shown in Supplementary Fig. S12B). **B** Control and NAXDko cells were grown in galactose without or with 5 mM inosine supplementation and indicated metabolites were quantified after 72 h by HPLC–UV. (NADPH and NAD^+^ levels are shown in Supplementary Fig. S12C.) **C** Control and NAXDko cells were grown without or with 5 mM inosine supplementation and indicated metabolites were measured after 72 h using ICMS and HILIC-MS (the complete mapped data are presented in Supplementary Fig. S13). Data were normalized using the summed MS1 intensities of all metabolites within each sample. All values are means ± SDs of at least three biological replicates. Statistical significance was calculated using an equal variance, unpaired Student’s *t*-test (*p < 0.05; **p < 0.01, ***p < 0.001; ****p < 0.0001; ns, not significant). Co, Control; Ko, NAXDKo; Gal, galactose; Ino, inosine; MS, mass spectrometry

Serine supplementation moderately increased the viability of HAP1 cells under galactose stress (Supplementary Fig. S12A). This effect did not depend on the serine concentration in the range that we tested (0.5–4 mM), and a similar relative effect was observed in control and NAXDko cells. The incomplete rescue by serine in the NAXDko cells indicates that other metabolic bottlenecks contribute to decreased growth on galactose in these cells. Interestingly, inosine supplementation led to a dose-dependent increase in cell viability in the galactose condition, with a more important relative effect in NAXDko cells than in control cells (Fig. 6A and Supplementary Fig. S12B). We analyzed the levels of normal and damaged NAD(P)H cofactors upon inosine supplementation by HPLC–UV (Fig. 6B and Supplementary Fig. S12C). As this treatment exerted only a moderate effect on the NADHX burden in NAXDko cells (Fig. 6B), we tried to gain further insight into the mechanism of action of inosine by performing more extensive metabolomic analyses of control and NAXDko cells cultivated under galactose stress without and with this supplement (Fig. 6C and Supplementary Fig. S13). As expected [33], in both control and NAXDko cells, inosine supplementation increased the intracellular levels of inosine, IMP, ribose-1-phosphate (product of inosine breakdown), and sedoheptulose-7-phosphate, and decreased the levels of upstream precursors of purine biosynthesis, such as phosphoribosyl pyrophosphate (PRPP), phosphoribosyl-*N*-formylglycineamide (FGAR), and 5-aminoimidazole-4-carboxamide ribonucleotide (AICAR) (Supplementary Fig. S13). More surprisingly, inosine supplementation strongly decreased the levels of the lower glycolytic triose phosphate intermediates as well as of 3-phosphoserine and serine, in both control and NAXDko cells (Fig. 6C). We also observed increased lactate levels (Fig. 6C) and decreased glycine levels (Supplementary Fig. S13) in both cell lines with inosine supplementation. As inosine directly fuels the purine salvage pathway, bypassing their de novo synthesis, which depends on serine-derived glycine and formate [39], it is possible that cells no longer depend (as much as they do in galactose stress alone) on de novo serine synthesis under inosine supplementation, which could rationalize at least part of the metabolic reprogramming as well as the more important rescue effect on the NAXDko cells observed upon this treatment.

**Fig. 7:**
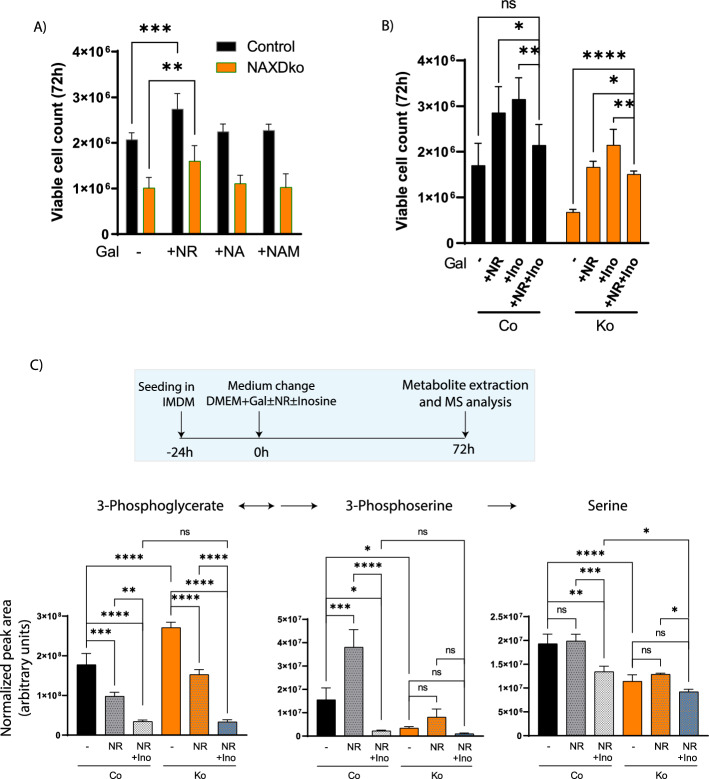
Nicotinamide riboside supplementation partially rescues growth and metabolic defects in NAXD-deficient HAP1 cells. **A** Control and NAXDko cells were grown in galactose without or with supplementation of NAD precursors (2 mM each), and viable cell count was measured using Acridine Orange and 4′,6-diamidin-2-phenylindole dyes after 72 h (this panel) or 96 h (Supplementary Fig. S14A; replots of the time course data relative to the control cell viability). **B** Control and NAXDko cells were grown without or with the indicated supplements (5 mM each), and viable cell count was measured after 72 h (replot of viable cell count in supplemented versus control conditions is shown in Supplementary Fig. S14D). **C** Control and NAXDko cells were grown in galactose without or with indicated supplements (5 mM each), and indicated metabolites were measured after 72 h by ICMS and HILIC-MS. Data were normalized using the summed MS1 intensities of all metabolites within each sample. All values are means ± SDs of 3–6 biological replicates. Statistical significance was calculated using an equal variance, unpaired Student’s *t*-test (**A**, **B**) or ordinary one-way ANOVA (**C**) (*p < 0.05; **p < 0.01, ***p < 0.001; ****p < 0.0001; ns, not significant). Co, Control; Ko, NAXDKo; Gal, galactose; NR, nicotinamide riboside; NA, nicotinic acid; NAM, nicotinamide mononucleotide; Ino, inosine; MS, mass spectrometry

Among the tested NAD precursors, only NR supplementation exerted a beneficial effect on growth in galactose condition for both the control and NAXDko cell lines (Fig. 7A and Supplementary Fig. S14A), probably owing to a reduced NADHX burden as observed in a preliminary experiment (Supplementary Fig. S14B). Mitochondrial superoxide staining with the MitoSOX dye also revealed a decrease of mitochondrial oxidative stress in galactose-grown control and NAXDko cells supplemented with NR, but not in cells supplemented with inosine (Supplementary Fig. S14C). The above observations with respect to inosine and NR suggested that both molecules exert their beneficial effects on NAXDko cells under galactose stress through different mechanisms and that their combined supplementation may exert additive effects by correcting growth and metabolic phenotypes. This hypothesis could, however, not be confirmed in our preliminary tests on galactose-grown control and NAXDko cells, as we observed that combined inosine and NR supplementation does not give a significant growth advantage in comparison with treatment with each supplement alone (Fig. 7B and Supplementary Fig. S14D).

To further explore the mechanism of action of NR (alone or in combination with inosine), we analyzed its effects on the serine synthesis pathway intermediates in control and NAXDko cells grown in galactose (Fig. 7C). With NR supplementation alone, decreased 3-phosphoglycerate and increased 3-phosphoserine levels were observed for both control and NAXDko cells, suggesting that NR increases the flux through the serine synthesis pathway. This may be explained by a partial relief of PHGDH inhibition due to a reduced NADHX burden (Supplementary Fig. S14B) and/or an increased NAD^+^/NADH ratio, previously shown to be an important modulator of the PHGDH-catalyzed step [29]. In contrast, combined supplementation of NR and inosine led to decreased levels of both 3-phosphoglycerate as well as 3-phosphoserine in both the control and NAXDko cells, suggesting that inosine supersedes the NR effect. A similar superseding effect of inosine was observed for serine levels (Fig. 7C). Overall, these metabolomic analyses indicated that the rescue mechanisms by which NR and inosine act are quite different, although they both involve the serine synthesis pathway. Under galactose stress, NADHX accumulation impairs cell growth at least partially by interfering with de novo serine synthesis, which can be counteracted by NR supplementation. Inosine exerts at least part of its rescue effect by relaxing the dependency of the galactose-grown cells on de novo serine synthesis by itself directly feeding into purine salvage.

## Inhibition of de novo serine synthesis is observed in NAXD patient-derived fibroblasts

**Fig. 8:**
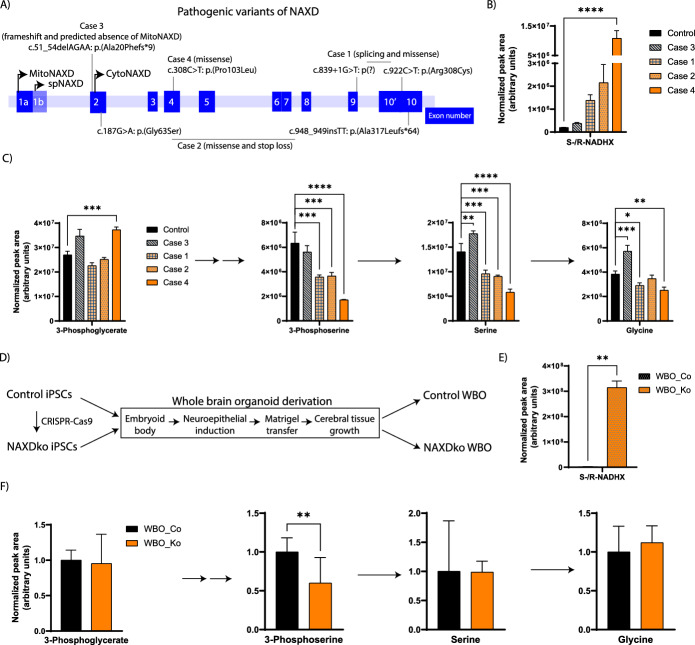
NAXD patient-derived fibroblasts and NAXDko whole brain organoids grown in galactose show impaired de novo serine synthesis. **A** Pathogenic mutations observed in patients are mapped on the *NAXD* gene. Case numbers are retained from the ones reported in Ref. [11], and further details on these cases are summarized in Table 1. Exons are shown as blue numbered boxes. **B**, **C** Control and patient fibroblasts were grown in galactose for 72 h, and NADHX (**B**) as well as serine synthesis pathway metabolites (**C**) were analyzed using ICMS or HILIC-MS methods. All values are means ± SDs of three independent replicates. Statistical significance was calculated using ordinary one-way ANOVA (*p < 0.05; **p < 0.01, ***p < 0.001; ****p < 0.0001). **D** Schematic representing WBO derivation from control and NAXDko human iPSCs. Control and NAXDko WBOs were grown in a rich medium for 75 days and shifted to a galactose containing medium for 72 h before analysis of NADHX (**E**) and serine synthesis pathway metabolites (**F**) by ICMS and HILIC-MS methods. Data were normalized using the summed MS1 intensities of all metabolites within each sample. For fibroblasts, values are means ± SDs of three biological replicates and statistical significance was calculated using ordinary one-way ANOVA. For WBOs, values are means ± SDs obtained from 8 samples (extracts), where each sample was obtained by pooling 2 organoids (the 16 organoids used in total per genotype were generated in 4 independent derivations). Statistical significance was calculated using an equal variance, unpaired Student’s *t*-test (*p < 0.05; **p < 0.01, ***p < 0.001; ****p < 0.0001). spNAXD, signal peptide NAXD; Co, Control; Ko, NAXDKo; WBO, whole brain organoid

Pathogenic mutations in the *NAXD* gene have been reported for more than ten individuals so far [12]. These mutations are found across the *NAXD* gene, and preliminary evidence suggests that mutations leading to loss of only the mitochondrial form of the enzyme yield a different clinical phenotype than mutations leading to the loss of both the mitochondrial and cytosolic enzyme forms [12]. In the light of the results described herein for NAXD-deficient HAP1 cells, suggesting de novo serine synthesis as a major target pathway for mediating pathogenic effects of NAXD deficiency, we wanted to test whether similar perturbations could also be observed in NAXD patient-derived fibroblasts. We measured NADHX and serine pathway metabolite levels in fibroblasts derived from four patients that all harbored different mutations in the *NAXD* gene (Fig. 8A and Table 1). One of the cases (case 3) carried a homozygous missense mutation predicted to lead to loss of only mitochondrial NAXD, while preserving expression of cytosolic NAXD. The mutations in the three other cases are predicted to affect expression of both the mitochondrial and cytosolic forms of NAXD. Fibroblasts from control and NAXD-deficient subjects were grown in galactose medium, and metabolites, extracted after 72 h of cultivation, were analyzed by ICMS and HILIC-MS. In agreement with previously reported measurements [11], we found NADHX to accumulate only at very moderate levels in fibroblast extracts of the “mitochondrial” NAXD case (case 3) while higher, although variable levels of the damaged cofactor were measured in the other cases (Fig. 8B). In good agreement with the HAP1 cell data, NAXD-deficient fibroblasts showed a tendency toward increased 3-phosphoglycerate and decreased 3-phosphoserine, serine, and glycine levels compared with control fibroblasts (Fig. 8C). Except for the mitochondrial NAXD case, the 3-phosphoserine and serine levels showed a negative correlation with NADHX levels. As for the HAP1 cells, we verified by western blot analysis that PHGDH protein levels are not decreased in fibroblasts derived from NAXD-deficient subjects compared with control fibroblasts (Supplementary Fig. S10B). These observations support the disease relevance of the metabolic phenotypes observed in our HAP1 cell model, at least for patients with whole-cell (mitochondrial and cytosolic) NAXD deficiency.

**Table 1:** Details of patient-derived fibroblasts used in the current study

NAXD case	Variant details	Effects of mutation(s)	Experimental evidence
Case 1	NM_001242882.1; c.839 + 1G > T: p(?) and c.922C > T: p.(Arg308Cys)	Splicing and missense	NADHX accumulation in patient-derived fibroblasts; recombinant p.(Arg308Cys) variant shows pronounced thermolability, reduced *V*_max_, and increased *K*_m_ compared with the wild-type protein [11]; reduced levels of respiratory complex I and mitoribosome subunits [19]
Case 2	NM_001242882.1; c.187G > A: p.(Gly63Ser) and c.948_949insTT: p.(Ala317Leufs*64)	Missense and frameshift with stop loss	NADHX accumulation in patient-derived fibroblasts; recombinant p.(Gly63Ser) variant shows pronounced thermolability, reduced *V*_max_, and increased *K*_m_ compared with the wild-type protein [11]; reduced levels of respiratory complexes I and IV as well as mitoribosome subunits [19]
Case 3	NM_001242882.1; c.51_54delAGAA: p.(Ala20Phefs*9)	Frameshift and nonsense-mediated decay	Lower NADHX accumulation in patient-derived fibroblasts [11]; absence of mitochondrially localized NAXD in HEK293FT cells transiently overexpressing the p.Ala20Phefs*9 variant [15]; reduced levels of respiratory complex I and mitoribosome subunits [19]
Case 4	NM_001242882.1; c.308C > T: p.(Pro103Leu)	Missense	NADHX accumulation in patient-derived fibroblasts; in silico analysis predicted the variant to be highly pathogenic [11]; reduced levels of respiratory complex I and mitoribosome subunits [19]

To generate a more complex model for NAXD deficiency, whole brain organoids (WBOs) were derived from a human iPSC line in which the *NAXD* gene was knocked out using CRISPR/Cas9 technology and following the protocol (Fig. 8D) from Lancaster and Knoblich [40]. A more detailed article on iPSC KO line generation and characterization of derived models will be published separately as it goes beyond the scope of the current manuscript. The control and NAXDko WBOs developed apparently normally (Supplementary Fig. S15). At the initial stages of organoid derivation, the embryoid bodies (EBs) were of comparable size (Supplementary Fig. S15A). Further during differentiation, we monitored the presence of neural progenitor cells (positive for SOX2 and PAX6), neurons (positive for MAP2), astrocytes (positive for GFAP and S100b), as well as oligodendrocyte progenitors (positive for Olig1 and Olig2). These different cell populations were observed in both control and NAXDko WBOs (Supplementary Fig. S15C). Also, no significant changes in the levels of extracellular glucose, glutamine, and lactate were observed during differentiation between the control and mutant WBOs (Supplementary Fig. S15B). On the basis of these results, we conclude that overall brain organoid development seems not to be substantially affected by NAXD loss of function (Supplementary Fig. S15). However, we observed a clear accumulation of NADHX in the NAXDko WBOs compared with control WBOs (Fig. 8E). Interestingly, although similar 3-PG and serine levels were measured in extracts of both control and NAXDko WBO organoids, we could detect a significant decrease in the NAXDko extracts in the levels of 3-phosphoserine, the product of the main target enzyme for NADHX inhibition identified in this study (Fig. 8F). These results indicate that serine pathway inhibition may also occur in certain brain cell types and could contribute to the neurological phenotype of NAXD-deficient patients.

## Discussion

**Fig. 9:**
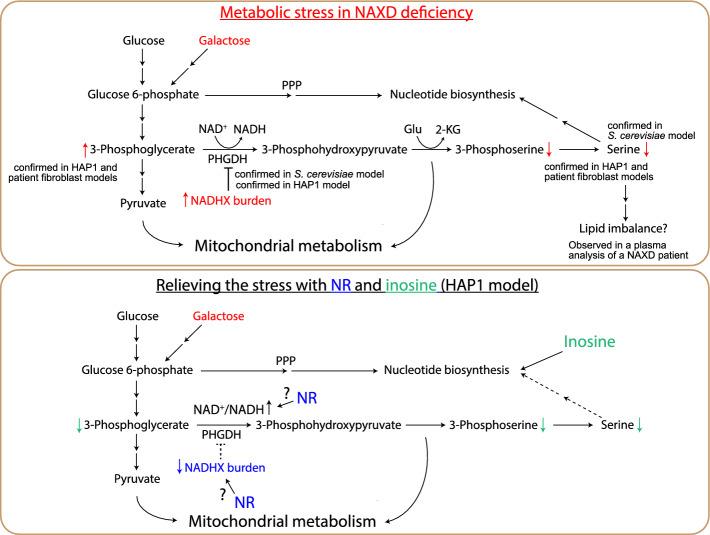
A working model summarizing key conclusions from this study. Upper panel: NAXD-deficient yeast and human cells display inhibition of de novo serine synthesis. In human cells, this effect is revealed only under certain conditions, such as cultivation in the presence of galactose instead of glucose. It is possible that this blockage of the serine synthesis pathway contributes to downstream perturbations in lipid metabolism, which were suggested on the basis of plasma metabolomic analyses in a NAXD patient [18]. Lower panel: NR and inosine partially rescue NAXD deficiency phenotypes via distinct mechanisms. Our results suggest that inosine exerts a rescue effect in HAP1 NAXDko cells by reducing the cells' reliance on the serine synthesis pathway for downstream metabolic processes, whereas NR supplementation presumably leads to positive effects via relieving PHGDH inhibition through reduced NADHX burden and/or increased NAD^+^/NADH ratio

Our previous work on a budding yeast model of NAXD deficiency [8] revealed inhibition of the serine pathway as a main metabolic perturbation resulting from loss of NAD(P)HX dehydratase function. Here, we found that this key downstream effect of NAXD deficiency in the yeast model is conserved in human HAP1 and patient-derived fibroblast cell models (Fig. 9). Using HAP1 NAXDko cells, we demonstrated this effect most clearly in cultivation media containing galactose instead of glucose as the carbon source, where the NAXDko cells also showed decreased viability. Although no difference in cell viability between NAXDko and control cells was observed in a standard, glucose-containing, IMDM medium, differences in steady-state levels of a number of metabolites, notably TCA cycle intermediates and monophosphonucleotides, were detected under those conditions. For the HAP1 NAXDko cells grown in galactose, strikingly different (and more pronounced) metabolic perturbations were observed than for the cells cultured in glucose. TCA cycle intermediates showed no significant differences between control and NAXDko cells grown in galactose, and of the mitochondrial parameters tested, only MitoSOX levels were subtly, but significantly, increased under galactose stress in the NAXDko cells compared with control cells. Using complementary ICMS and HILIC-MS methods that provide great coverage of central cytosolic metabolic pathways, we found prominent differences in steady-state levels of serine synthesis pathway intermediates, with accumulation of 3-phosphoglycerate and decreased levels of 3-phosphoserine and serine in NAXDko cells (3-phosphohydroxypyruvate was not detected in our metabolomic analyses). These metabolic signatures and our earlier work on the yeast model [8] led us to hypothesize that PHGDH, the first enzyme in the serine biosynthesis pathway, may be inhibited by NADHX, and this was indeed confirmed by stable isotope labeling analyses in living cells and by direct in vitro assays of purified human PHGDH. For the tracer analyses, in particular pulse labeling with ^13^C_6_-glucose in the galactose-grown cells, an approach which has to our knowledge not been reported before, allowed us to demonstrate a clear decrease of label incorporation into 3-phosphoserine in the NAXDko cells compared with control cells. These observations show that metabolic phenotypes in NAXD-deficient cell models can result from perturbations in different subcellular compartments that are more or less pronounced depending on the cultivation conditions.

Although we had previously found that yeast PHGDHs are potently inhibited by NADHX [8], we did not a priori expect to observe this effect with human PHGDH, given the marked differences in catalytic mechanisms recently demonstrated for the enzymes from both species [29]. Whereas the yeast PHGDHs are transhydrogenases tightly binding NAD, acting as an intermediate electron acceptor during the oxidation of 3-PG (the final electron acceptor being 2-ketoglutarate), human PHGDH acts as a classical dehydrogenase, releasing the NAD cofactor after reduction. As our unbiased metabolomic and stable isotope tracing analyses clearly indicated inhibition of the first step of serine biosynthesis in living NAXDko cells, we assayed the dehydrogenase activity of recombinant, purified hPHGDH in the presence of different forms of NADHX. As preliminary inhibition assays with human PHGDH in the presence of saturating substrate (3-PG) concentrations did not reveal any inhibition by NADHX [29], we tested the inhibition at lower (near *K*_m_) 3-PG substrate concentrations (Fig. 4B). Under those conditions, we found that, similar to the yeast enzyme, *S*-NADHX inhibits the activity of the human enzyme more potently than the *R*- or cyclic forms. The inhibitory effect on the yeast enzymes was, however, more potent, with almost complete inhibition achieved at sub-micromolar concentrations of *S*-NADHX [8]; 100-fold higher concentrations were needed to achieve a similar effect for human PHGDH (Fig. 4B). This difference is most likely related to the differences in catalytic properties mentioned above for the yeast and human enzymes. Our enzymatic activity assays in extracts of NAXDko cells did not point to any posttranslational modifications of the PHGDH enzyme as a consequence of NADHX repair deficiency. Taken together, our observations strongly suggest that PHGDH is directly inhibited by the increased levels of NADHX in NAXD-deficient human cells and that this effect is sufficient to markedly reduce the flux through the serine synthesis pathway under nutritionally challenging conditions, such as growth in galactose medium.

Concerning the use of alternative hexose sugars (other than glucose) for human cell cultivation, it was recently shown that acute myeloid leukemia cells grown in high-fructose conditions are more dependent on the serine biosynthesis pathway (compared with cells grown in glucose), and that pharmacological inhibition of PHGDH markedly reduces their growth [41]. It is also known that cancer cells become progressively reliant on glutamine consumption for energy production when switched from glucose to galactose or fructose [30], fueling 2-ketoglutarate formation through the serine synthesis pathway at the level of PSAT1 and allowing for proliferation even in the absence of glucose [41]. In another recent study, Benzarti et al. [42] provide additional evidence for the importance of the serine synthesis pathway in cancer cells cultivated in the presence of galactose instead of glucose. The presence of galactose induces important metabolic rewirings allowing to maintain sufficient flux in the serine synthesis pathway despite reduced glycolytic flux. This metabolic reprogramming includes diversion of galactose carbon toward serine synthesis by blocking glycolysis at the level of PKM2 and redirection of glutamine metabolism toward serine synthesis via mitochondrial PEPCK2. Taken together, these observations suggest that many cancer cell lines become more reliant on the serine synthesis pathway when cultivated on galactose instead of glucose and can explain why specific growth and metabolic phenotypes of HAP1 NAXDko cells were revealed specifically under this condition. While galactose exposure of cultured cells induces glycolytic limitation, mimicking what can for instance occur in vivo at the core of bigger tumors owing to progressive glucose depletion [42], it is also worth noting that galactose derived from nutrition can be taken up and metabolized by healthy organs, with notably its active conversion to amino acids, including serine, in the brain [43].

Another striking feature of HAP1 cells grown in galactose medium, as shown previously and also observed in this study, is an increased NAD^+^/NADH ratio compared with cells grown in glucose medium, mostly owing to increased respiratory activity [31]. As NAD^+^ and NADH levels were comparable, however, in the galactose condition between control and NAXDko cells, despite increased damaged NADH levels in NAXDko cells, we surmise that the latter maintain healthy cofactor levels by increasing the flux of de novo NAD synthesis and/or salvage pathways. The increased levels of NADHX in NAXDko cells grown in galactose medium compared with cells grown in glucose medium suggest that the galactose condition accelerates NADHX formation by enzymatic and/or nonenzymatic reactions that remain to be identified.

We attempted to correct the metabolic phenotype in NAXDko cells by genetic means and by supplementation of small molecules. Cytosolic expression of NAXD in NAXDko cells alleviated NADHX burden and resulted in a near-complete rescue of 3-phosphoglycerate and serine levels. Restoring expression of the mitochondrial form of NAXD resulted in a much more partial rescue in 3-phosphoglycerate and serine levels, strongly indicating that cytosolic NADHX clearance is most effective at relieving inhibition of PHGDH. A review of the clinical features of all known NAXD patients suggested that one can distinguish between two forms of the disease [12]: a first form where NAXD mutations will lead to loss of both the cytosolic and mitochondrial expression of NAXD (best modeled by our NAXDko cell line), with a clinical picture dominated by severe neurological symptoms and skin lesions, and a second form of the disease that seems to manifest when mutations lead to defective mitochondrial NAXD targeting, with residual cytosolic activity but no repair activity within the mitochondria (best modeled by our CytoNAXD rescue line). In the latter disease form, patients present with predominant myopathy symptoms and no skin lesions. These observations combined with our metabolomic findings in the NAXDko and the CytoNAXD rescue lines would support that neurological and skin manifestations observed in whole cell deficiency of NAXD are caused primarily by perturbed serine synthesis and/or other perturbed cytosolic pathways that remain to be uncovered, whereas muscle pathology will predominate when NADHX accumulates mostly in the mitochondria.

For phenotypic rescue assays in the NAXDko cells under galactose condition with small-molecule supplementation, we selected three compounds on the basis of previous literature and our metabolic profiling: serine, inosine, and NR. Serine supplementation did not relieve the growth inhibition of NAXDko cells in galactose, indicating that other metabolic bottlenecks limit cell growth under this condition. It is interesting to note that others also observed that the phenotypes resulting from PHGDH deficiency (reduced cell growth or metabolic imbalances) or its inhibition are not always rescued by supplementation with serine [35, 44, 45] and, in some cases, supplementation with 2-ketoglutarate plus ribose showed a better rescue effect [46]. The enzyme downstream of PHGDH is a transaminase (PSAT1) that catalyzes formation of 3-phosphoserine by transfer of an amino group from glutamate (derived from glutamine in the medium) [30, 35], leading to consumption of glutamate, which is critical for cells cultured in galactose [30], and production of 2-ketoglutarate, which is further utilized by mitochondrial metabolism [35]. Simple supplementation of serine may therefore not compensate for other metabolic limitations resulting from PHGDH inhibition.

Supplementation with NR or inosine exerted a partial and comparable growth rescue effect but had distinct impacts on serine pathway intermediates. Inosine supplementation, which stimulated growth of both control and NAXDko cells grown in galactose, resulted in dramatically reduced levels of lower (starting from 1,3-bisphosphoglycerate) glycolytic intermediates (except pyruvate) and all serine pathway metabolites (Fig. 6 and Supplementary Fig. S13), indicating that, in the presence of inosine, these cells do not depend on an active serine synthesis pathway. Interestingly, previous work on isolated mouse pancreatic islets also indicated reduced levels of lower glycolytic intermediates upon inosine supplementation [47], but the molecular mechanism underlying this “inosine” effect remains elusive. By contrast, NR supplementation induced decreased levels of 3-phosphoglycerate and increased levels of 3-phosphoserine compared with untreated cells, indicating relief of PHGDH inhibition, possibly due to an increase of the NAD^+^/NADH ratio [38] and reduced NADHX burden. Our metabolic analyses clearly indicate that both inosine and NR operate through distinct mechanisms to impart their beneficial effect on NAXDko cells (Fig. 9, lower panel), but more work is required to further elucidate their precise mechanism of action.

To explore the translational value of our HAP1 cell-based findings, we tested whether inhibition of the de novo serine synthesis pathway could also be observed in NAXD patient-derived fibroblasts. By measuring steady-state levels of the pathway intermediates, we provide strong evidence that this pathway is also inhibited at the level of PHGDH in NAXD patient fibroblasts, indicating that this metabolic perturbation could be involved in the pathophysiology of the disease (see below). Interestingly, the fibroblasts from the patient (case 3) with a NAXD mutation predicted to only prevent mitochondrial NADHX repair activity (and not cytosolic repair activity) did not show decreased serine synthesis pathway activity based on these measurements, further supporting that, in this form of the disease, the predominant myopathy symptoms are caused by perturbed mitochondrial metabolism. Patient fibroblasts shifted to basal medium with galactose could be grown in the presence of dialyzed FBS, resulting in a culture medium composition that was even more challenging for growth than the one used for HAP1 cell experiments (where nondialyzed FBS had to be used). This probably allowed us to detect serine pathway inhibition in the patient-derived fibroblasts despite variable levels of residual NAXD function (as opposed to our NAXD null model in the HAP1 cells). Tissue-specific expression of the different NAXD isoforms could also contribute to the phenotypic heterogeneity in NAXD patients. According to the GTEx portal, the *NAXD* gene is expressed ubiquitously, with highest bulk expression values found in the cerebellum and the ovary. Unfortunately, no information on the tissue distribution of the most abundant transcript variant (NM_001242882; encoding the mitochondrial and cytosolic *NAXD* isoforms [11]) is available in GTEx.

Whole brain organoids derived from NAXDko human iPSCs developed apparently normally, but showed high NADHX accumulation compared with control organoids as well as decreased levels of the PHGDH product 3-phosphoserine, indicating that high NADHX levels also interfere with de novo serine synthesis in whole brain organoids. It is likely that there are brain cell type-specific phenotypes yet to be identified. This forms a promising basis to further exploit this disease-relevant, and much more complex, organoid model for NAXD deficiency research.

In all patients described so far, febrile illness or other stress triggers have initiated a rapid irreversible clinical decline with premature death, except for two NAXD [14, 18] and three NAXE [13, 23, 26] cases. The patients who did not succumb after neurological crisis episodes had all received oral administration of the NAD precursor niacin (vitamin B3). In a recent study [18], plasma samples from a NAXD patient were analyzed at the time of crisis and after niacin treatment. In addition to perturbed nicotinamide metabolism at the time of crisis, the authors also found that the levels of plasmalogens, phospholipids, and lysophospholipids were altered, and speculated that these changes could be a consequence of impaired mitochondrial function and/or disturbed serine metabolism. Another study on a NAXE patient revealed similar perturbations in levels of phospholipids, diacylglycerols, and saturated and unsaturated fatty acids as well as plasmalogens at the time of crisis [13]. It should be noted that most of these perturbations in the NAXD and NAXE patients were restored after niacin therapy for 6 months [13, 18]. Metabolic profiling of cardiac tissue of a NAXE-deficient mouse model recently showed increased levels of the lipidic molecules cholesterol, α-linolenic acid, and deoxycholic acid [48]. Interestingly, systemic metabolic profiling of the NAXE deficiency mouse model showed reduced levels of complex lipids, phospholipids, sphingomyelin, and plasmalogens [28].

The brain is highly dependent on de novo synthesis of serine (owing to poor blood–brain barrier penetration of serine), and utilizes it for synthesis of downstream metabolites, such as glycine, D-serine, sphingolipids, and phospholipids [49]. Accordingly, primary serine biosynthesis defects lead to a phenotype with a broad spectrum of severity, but always characterized by predominant central nervous system abnormalities. It is perhaps not surprising, given the metabolic fates of serine, that perturbed phospholipid metabolism has recently been demonstrated in those patients [50]. Patients with milder forms of the disease also develop progressive peripheral neuropathy [51]. Interestingly, serine deficiency has in recent years been linked with additional rare disorders (e.g., inherited mitochondrial disorders [51]) and chronic medical disorders (e.g., diabetes mellitus [52]) associated with peripheral neuropathy, and increasing evidence converges on the idea that toxic deoxysphingolipids accumulating as a consequence of serine deficiency drive the pathological changes in peripheral nerves [53]. Inhibition of serine biosynthesis is observed in our yeast [8], HAP1, and patient-derived fibroblast models (current study) of NAXD deficiency, and its downstream metabolites (serine-derived lipids) showed modulated levels in NAXD and NAXE patient plasma samples and tissue samples of a NAXE-deficient mouse model [18, 48]. Taken together, we speculate that this disturbed lipid metabolism results at least in part from perturbations in the serine biosynthetic pathway in NAXD deficiency (Fig. 9, upper panel) and that it could play a role in the disease pathogenesis, in addition to other effects resulting from PHGDH inhibition (decreased formation of cytosolic 2-ketoglutarate, decreased availability of serine as a precursor for other important molecules) and perturbations in non-serine-related metabolic pathways that remain to be discovered. In the light of accumulating evidence linking serine deficiency with perturbed sphingolipid metabolism and peripheral neuropathy, it will be important to measure these lipid species, including deoxysphingolipids, in NAXD and NAXE patients and examine for evidence of peripheral neuropathy, at least in milder forms of the disease (which exist, since isolated adult cases have been reported [18, 19, 23]). Even though serine did not rescue the growth of galactose-grown HAP1 cells, our results suggest that serine supplementation in NAXD (and potentially also NAXE) patients could have beneficial effects, and this could first be tested in preclinical studies on whole organism models of NAXD deficiency. This is also supported by the beneficial effects demonstrated for serine supplementation in patients with primary deficiencies in de novo serine synthesis [54–58].

## Conclusions

We found that HAP1 NAXDko cells, as previously shown for NAXD patient-derived fibroblasts [11], exhibit reduced viability specifically when cultured in galactose medium. Through comprehensive analyses—including untargeted metabolomics, stable isotopic tracing, and in vitro biochemical assessments—we demonstrate that the lack of functional NAXD in human cells inhibits serine biosynthesis via the accumulation of NADHX, a damaged form of NADH found in NAXD- (and to a lesser extent NAXE-) deficient patients. This inhibition is mediated by PHGDH, as illustrated in Fig. 9. We propose that the stronger dependence of many cell lines on the serine synthesis pathway in galactose culture conditions clarifies why more pronounced phenotypes emerged in our NAXDko model under these conditions.

Furthermore, our research identifies serine and inosine as potential supplements to enhance the therapeutic efficacy of niacin in NAXD patients (Fig. 9). Our data also suggest that NR could serve as an alternative therapeutic approach, given its superior rescue effect in NAXDko cells and its reported higher efficiency as an NAD precursor compared with niacin in humans [59]. Altogether, these insights deepen our understanding of NAXD-related metabolic dysfunctions and open avenues for targeted therapeutic strategies that may improve the clinical management of NADHX repair deficiencies.

## Materials and Methods

### Materials

All reagents were purchased from Sigma Aldrich (Belgium) and were of analytical or cell culture grade, if not indicated otherwise. NR chloride was procured from MedKoo Biosciences, Inc. (catalog no. 329479).

## Culture of HAP1 cells and patient-derived fibroblasts

The HAP1 control (catalog no. C631), NAXDko (catalog no. HZGHC002518c011), and NAXEko (catalog no. HZGHC002520c002) cell lines were procured from the Horizon Discovery Group (Austria). The knock-out lines were generated using CRISPR/Cas9 technology and confirmed using Sanger sequencing [8]. The HAP1 cells were maintained in a medium containing Iscove’s modified Dulbecco’s medium (IMDM; 21,980,032, Gibco-Thermo Fisher Scientific) with 10% FBS (10,500,064, Gibco-Thermo Fisher Scientific), 100 units/mL penicillin, and 100 µg/mL streptomycin (15,070,063, Gibco-Thermo Fisher Scientific) at 37 °C with 5% CO_2_. The cells were passaged thrice a week before they reached approximately 80% confluency.

The patient-derived fibroblasts were obtained as described previously [11], and the same case numbering was maintained in this study. Detailed case descriptions can be found in the prior publication [11]. Some of the key features of these cases are summarized in Fig. 8A and Table 1. Primary cultures of fibroblasts from patients and age-matched controls were maintained in a medium containing high-glucose DMEM (11,965,092, Gibco-Thermo Fisher Scientific) with 10% FBS, 100 units/mL penicillin, and 100 µg/mL streptomycin at 37 °C with 5% CO_2_. The cells were passaged, with a medium change, once or twice a week when they reached confluency.

## Cell seeding, medium changes, and live cell counting

For cell culture experiments, typically 2.5 × 10^5^ cells (HAP1 or fibroblasts) were seeded per well into standard six-well plates (140,685, Nunclon Delta Surface, Thermo Fisher Scientific). Viable cell counting was performed with a Nucleocounter NC-3000 (910–3013, ChemoMetec, Germany), using Solution 13 (containing a mixture of Acridine Orange and 4′,6-diamidin-2-phenylindole dyes; ChemoMetec) according to the manufacturer’s instructions.

For medium changes, the cells (HAP1 or fibroblasts) were grown for 24 h in their respective maintenance medium (IMDM for HAP1 and high-glucose DMEM for fibroblasts) and were then carefully washed with prewarmed (37 °C) phosphate-buffered saline. After addition of the desired basal medium (prewarmed to 37 °C), cells were kept at 37 °C with 5% CO_2_ for variable durations depending on the design of the experiment. The basal medium for HAP1 cells contained DMEM5030 (D5030, Sigma Aldrich) with 10% FBS, 4 mM glutamine, 25 mM glucose or galactose, 100 units/mL penicillin, and 100 µg/mL streptomycin. The basal medium for fibroblasts contained DMEM5030 without serine/glycine and was supplemented with 10% dialyzed FBS (26,400,044, Gibco-Thermo Fisher Scientific), 4 mM glutamine, 25 mM galactose, 100 units/mL penicillin, and 100 µg/mL streptomycin.

For stable isotope labeling experiments, HAP1 cells were cultured in IMDM for 24 h and then shifted to the basal medium containing galactose. Stable isotopes were added at the indicated time points, depending on the design of the experiment (shown as schematics in the respective figures). For ^13^C_5_-glutamine (CLM-1166, Cambridge Isotope Laboratories) and ^13^C_6_-galactose (GAL-013, Omicron Biochemicals) labeling, the basal medium lacked the respective nonlabeled metabolites to avoid dilution of the label. The ^13^C_6_-glucose (CLM-1396, Cambridge Isotope Laboratories) was directly spiked into the cell culture in (nonlabeled) galactose-containing basal medium, followed by metabolite extraction 10 min or 30 min later.

## Metabolite extractions

For metabolomic experiments in galactose stress, to achieve a similar number of cells at the time of extraction, HAP1 NAXDko (or NAXDko-eGFP) cells were seeded into standard six-well plates (140,685, Nunclon Delta Surface, Thermo Fisher Scientific) at a density of 5 × 10^5^ cells/well whereas HAP1 control and all other cell lines were seeded at a density of 2.5 × 10^5^ cells/well (Supplementary Fig. S7A). For experiments in IMDM or high-glucose DMEM medium, all cell lines were seeded at 2.5 × 10^5^ cells/well.

Cells were gently washed either with prewarmed (37 °C) 100 mM HEPES (pH unadjusted; for ICMS measurements) or with prewarmed phosphate-buffered saline (for all other metabolite measurements). Metabolites were then extracted with addition of 750 µL precooled (-20 °C) extraction fluid (4:1 methanol/20 mM Tris, pH 8 for HPLC–UV measurements, or 4:1 methanol/water for other measurements; methanol, AE71.2, Carl Roth) into the wells and immediately scraping cells using a cell scraper. The collected extracts were kept in Eppendorf tubes placed on ice. To recover a maximum of material, another 250 µL of extraction fluid was added to each well and mixed with the original extract. The pooled extract was then centrifuged at 21,000*g* for 5 min at 4 °C. The resulting pellets were kept for normalization purposes (after protein quantification), whereas the supernatants (625 µL) were added to 2-mL Eppendorf tubes prefilled with 500 µL chloroform (7331.2, Carl Roth) at 4 °C, followed by addition of 325 µL of 20 mM Tris, pH 8 (4 °C; for HPLC–UV measurements) or water (4 °C; for other measurements). After a vigorous vortex (30 s to 1 min for HPLC–UV measurements or 20 min at 4 °C for other measurements), the extracts were centrifuged at 21,000*g* for 5 min at 4 °C. The upper polar phase was then filtered through a PHENEX-RC 4-mm syringe filter and lyophilized overnight. The samples were either used directly for metabolite analysis or stored at −80 °C until analysis.

For metabolite extractions from whole brain organoids, two organoids were pooled per sample in a Precellys tube, washed twice with Milli-Q water, and immediately plunged into liquid N_2_. Prechilled ceramic beads (600 mg) were added along with 750 µL of extraction fluid (−20 °C), and samples were homogenized for 30 s at 6000 rpm using a bead mill homogenizer system (Precellys24), maintaining a temperature of about 0 °C. The beads were allowed to settle, and extracts were transferred to a fresh prechilled tube. The beads were washed with additional 150 µL of extraction fluid (−20 °C), which were then pooled with the original extract. The pooled extract was centrifuged at 21,000*g* for 5 min at 4 °C. The resulting pellets were kept for normalization purposes (after protein quantification), whereas the supernatants (625 µL) were added to 2-mL Eppendorf tubes prefilled with 500 µL chloroform (4 °C), followed by addition of 325 µL of water (4 °C). After a vigorous vortex (20 min), the extracts were centrifuged at 21,000*g* for 5 min at 4 °C. The upper polar phase was filtered through a PHENEX-RC 4-mm syringe filter and lyophilized overnight. The samples were either used directly for metabolite analysis or stored at −80 °C until analysis.

## Metabolite measurements

### Targeted NAD(P)(H)(X) measurements by HPLC–UV

The HPLC–UV method for measurement of NAD(P)(H)(X) levels was adapted from Becker-Kettern et al. (2018) [8]. In short, the lyophilized extracts were analyzed immediately after resuspension in 15 µL of 10 mM Tris (pH 8) on a Shimadzu Nexera UHPLC system equipped with a photodiode array and fluorescence detectors. Samples (10 µL) were injected onto a Polaris C18-A column (3 × 150 mm, 3 µm particle size, Agilent, Belgium) connected to a SecurityGuard ULTRA C18 precolumn (for 3-mm internal diameter columns, Phenomenex). Ammonium acetate (84,885.180P, VWR) at 50 mM and pH 7.0 was used as mobile phase, and compounds were eluted with acetonitrile (ACN; AE70.2, Carl Roth) using the following gradient: 0 min, 0% ACN; 20 min, 5% ACN; 25 min, 7.5% ACN; 32 min, 90% ACN; 33 min, 0% ACN; 40 min, 0% ACN (re-equilibration step). The NADHX standards used for the HPLC and mass spectrometry (see below) analyses were prepared in-house as described previously [8]. The NAD(P)H concentrations were calculated from their absorbance at 340 nm using an extinction coefficient of 6220 M^−1^ cm^−1^. The *R*-, *S*-NADHX concentrations were calculated from their absorbance at 290 nm using an extinction coefficient of 13,500 M^−1^ cm^−1^ [60].

### Targeted NAD(P)(H)(X) measurements by LC–MS

Polar cell extracts were analyzed using an Agilent 1290 LC coupled to an Agilent 6560 Q-TOF MS equipped with a Dual Agilent Jet Stream ESI source. The analytical column (Polaris 180 Å C18-A, 3 × 150 mm, 3 µm) was maintained at 25 °C. The autosampler was kept at 4 °C, and the injection volume was 5 µL. The mobile phases consisted of 50 mM ammonium acetate in Milli-Q water (18.2 MΩ•cm, < 3 ppb TOC; eluent A) and acetonitrile (eluent B), and the flow rate was set to 0.2 mL/min. A linear gradient from 1% to 5% eluent B over 22 min was applied, followed by a linear increase to 99% eluent B in 5 min. After 3 min, eluent B was decreased to 1% (starting condition) within 0.5 min followed by re-equilibration in this condition for another 9.5 min. MS analysis was performed using electrospray ionization in positive mode (+ESI, capillary voltage of 3 kV, nozzle voltage of 1 kV). The protonated molecules were monitored in high-resolution mode (slicer position: 5) and extended dynamic range (2 GHz) with the following Q-TOF MS conditions: drying gas temperature, 325 °C; drying gas flow, 10 L/min (nitrogen); nebulizer, 35 psig; sheath gas temperature, 350 °C; sheath gas flow, 11 L/min; fragmentor, 400 V; Oct RF Vpp, 750 V. Full scan spectra were acquired for an *m*/*z* range of 100 to 1700 (0.5 spectra/s). External mass calibration was performed before measurement of each set of samples. In addition, a reference solution was used for online mass correction during the acquisition. All data were acquired with Agilent Mass Hunter LC/MS Data Acquisition (version B.08.00). Agilent Mass Hunter Qualitative Analysis (version B.08.00 SP1) was used for peak annotation and integration of the protonated molecules (Table 2).

**Table 2:** Targeted NAD(H)(X) ions and their retention times

ID	Exact mass (u)	[M + H]^+^ (m/z)	[M-H_2_O + H]^+^ (m/z)	Sum formula	Retention time (min)
NAD^+^	664.1169	(665.1242)*		C21 H28 N7 O14 P2	10.77
NADH	665.1248	666.132		C21 H29 N7 O14 P2	14.06
*S*-NADHX	683.1353		666.132	C21 H31 N7 O15 P2	9.68
*R*-NADHX	683.1353		666.132	C21 H31 N7 O15 P2	11.64
cNADHX_1	665.1248	666.132		C21 H29 N7 O14 P2	17.11
cNADHX_2	665.1248	666.132		C21 H29 N7 O14 P2	18.76

^*^Doubly charged ion was used for quantification

### Derivatization and untargeted GC–MS measurements

Metabolite derivatization was performed by using a multipurpose sample preparation robot (Gerstel). Dried polar cell extracts were dissolved in 20 µL pyridine (270,970, Sigma Aldrich), containing 20 mg/mL methoxyamine hydrochloride (226,904, Sigma Aldrich), and incubated for 120 min at 45 °C under shaking. After adding 20 µL *N*-methyl-*N*-trimethylsilyl-trifluoroacetamide (MSTFA; 701,270.510, Macherey–Nagel), samples were incubated for an additional 30 min at 45 °C under continuous shaking.

GC–MS analysis was performed by using an Agilent 7890B gas chromatograph (GC) coupled to an Agilent 5977A inert XL Mass Selective Detector (MSD). A sample volume of 1 µL was injected into a Split/Splitless inlet, operating in splitless mode at 270 °C. The GC was equipped with a 30 m (internal diameter 0.25 mm, film 0.25 µm) ZB-35 ms capillary column (Phenomenex) with a 5-m guard column in front of the analytical column. Helium was used as carrier gas with a constant flow rate of 1.2 mL/min. The GC oven temperature was held at 90 °C for 1 min and then increased to 270 °C at 9 °C/min. Then, the temperature was increased to 320 °C at 25 °C/min and held for 7 min. The total run time was 30 min. The transfer line temperature was set to 280 °C. The MSD was operating under electron ionization at 70 eV. The MS source was held at 230 °C, and the quadrupole at 150 °C. Mass spectra were acquired in full scan mode (*m*/*z* 70–700).

All GC–MS chromatograms were processed using MetaboliteDetector (v3.220190704) [61]. Compounds were automatically annotated by retention index and mass spectrum using an in-house mass spectral library. Retention index calibration was based on a C10–C40 even *n*-alkane mixture (68,281, Sigma Aldrich). The following deconvolution settings were applied: peak threshold, 10; minimum peak height, 10; bins per scan, 10; deconvolution width, 5 scans; no baseline adjustment; minimum 15 peaks per spectrum; no minimum required base peak intensity. The internal standard U-^13^C-ribitol (ALD-062, Omicron Biochemicals) was added at the same concentration to every sample to correct for uncontrolled sample losses and analyte degradation during metabolite extraction. The dataset was normalized by using the response ratio of the integrated peak areas (analytes/U-^13^C-ribitol).

### Untargeted LC–MS/MS analysis

Polar cell extracts were analyzed using an Agilent 1290 LC coupled to an Agilent 6560 Q-TOF MS equipped with a Dual Agilent Jet Stream ESI source. The analytical column (Kinetex EVO 100 Å C18, 2.1 × 150 mm, 2.6 µm) was maintained at 30 °C. The autosampler was kept at 4 °C, and the injection volume was 5 µL. The mobile phases consisted of Milli-Q water (18.2 MΩ•cm, < 3 ppb TOC) with 0.1% formic acid (84,865.180, VWR) as eluent A and acetonitrile:methanol (1:1) with 0.1% formic acid as eluent B, and the flow rate was set to 0.25 mL/min. An isocratic flow with 5% eluent B was maintained for the first 5 min, linearly increased to 98% B from 5 to 20 min, followed by a linear decrease to 5% B within 0.1 min. The column was re-equilibrated with 5% B for 5 min.

MS and MS/MS analysis were performed using electrospray ionization in negative mode (−ESI, capillary voltage of 2 kV, nozzle voltage of 0.5 kV) and positive mode (+ESI, capillary voltage of 3 kV, nozzle voltage of 1 kV) in two separate LC–MS/MS runs. The charged molecules were monitored in high-resolution mode (slicer position: 5) and extended dynamic range (2 GHz) with the following Q-TOF MS conditions: drying gas temperature, 325 °C; drying gas flow, 10 L/min (nitrogen); nebulizer, 35 psig; sheath gas temperature, 350 °C; sheath gas flow, 12 L/min; fragmentor, 380 V; Oct RF Vpp, 750 V. Full scan spectra were acquired for an *m*/*z* of 100–1700 (0.5 spectra/s). Scan range for auto MS/MS acquisition was 100–1100 *m*/*z* (0.5 spectra/s) for the MS scan and 50–1100 *m*/*z* (0.25 spectra/s) for MS/MS scans in information-dependent acquisition mode. Precursor selection window was 1.3 *m*/*z*, and collision energy was −40 V with ±30 spread. External mass calibration was performed before measurement of each set of samples. In addition, a reference solution was used for online mass correction during the acquisition. Precursor charge state was set to “All” mode, intensity threshold of 1000 cps, mass tolerance 20 ppm, and Active Exclusion was enabled with exclusion after 1 spectra during 0.13 min. All data were acquired with Agilent Mass Hunter LC–MS/MS Data Acquisition (version B.08.00). Agilent “.d”-raw files were converted to the mzML file format using the Proteowizard software package (version 3.0.22182). Data analysis was performed using MS-DIAL (version 5.1.221218) using default settings for high-resolution LC–MS data with soft ionization, except that the retention time window was set to 2 min. Before final data analysis, the following *m*/*z* features were removed: features without an assigned annotation, features without an assigned chemical formula, features with halogens in their assigned formula, and features assigned as pesticides.

### ICMS measurements

For the ICMS measurements, polar extracts were analyzed as previously described [33] with the following adjustments. Dried polar metabolite pellets were reconstituted in 25 µL of nanopure water (Milli-Q Advantage A10), and an injection volume of 20 µL was used. For the ICMS system, we interfaced a Dionex ICS-6000+ ion chromatograph with a QExactive high-resolution mass spectrometer (both Thermo Fisher Scientific). The guard and main columns, gradient settings, and suppressor type remained unchanged [33]. The heated electrospray ionization settings were as follows: spray voltage of 2.500 kV, auxiliary gas temperature of 420 °C, sweep gas flow rate of 0, auxiliary gas flow rate of 15 units, and sheath gas flow rate of 50 units. The settings for acquisition of MS1 data were: *m*/*z* scan of 80–750, resolution of 17.5 K, automatic gain control (AGC) target of 1 × 10^6^, and maximum injection time of 100 ms. Data were acquired using two micro-scans. For the MS2 data acquisition, the following settings were used: resolution of 17.5 K, AGC of 2 × 10^5^, maximum injection time of 50 ms, loop count 2, isolation window of 4.0 *m*/*z*, and collision energy of 30 eV. For stable isotope labeling (SIL) analyses, only MS1 ion intensities were acquired. Peak areas were integrated and exported to Microsoft Excel (RRID: SCR_016137) via the Thermo TraceFinder software (version 5.1; RRID: SCR_023045). Metabolite identification confidence was mostly level 1 [62]. The confidence levels together with raw and normalized peak intensity values for all mass spectrometry measurements are provided in Additional file 1 (non-SIL data) and Additional file 2 (SIL data). The non-SIL data were normalized either using the summed MS1 intensities of all metabolites within each sample or by protein amounts, as indicated. For the SIL data, we performed correction for naturally occurring ^13^C and ^13^C fractional enrichment calculation using the IsoCor software (version 2.2.0) [63].

### HILIC-MS measurements

Reconstituted dried polar cell extracts were analyzed using a Themo UHPLC Vanquish UHPLC equipped with a quaternary pump and coupled to a Thermo Q Exactive HF mass spectrometer. Metabolites were separated using a HILIC column (SeQuant ZIC pHILIC, 5 μm particles, 2.1 × 150 mm). The column oven temperature was set at 45 °C, and the autosampler was maintained cooled at 4 °C. Mobile phases, applied at a flow rate of 0.2 mL/min, consisted of Milli-Q water (mobile phase A) and acetonitrile:water (90:10, v:v; mobile phase B), both with 20 mM ammonium acetate and 5 µM medronic acid (5191–4506, Agilent) added. The gradient was as follows: isocratic 100% B, 0–2 min; linear decrease to 22.2% B, 2–17.5 min, isocratic 22.2% B, 17.5–19 min; linear increase to 100% B, 19–20 min; isocratic 100% B, 20–31 min. Samples were reconstituted in mobile phase B, and an injection volume of 10 µL was used. For the MS analysis, we used the following settings: sheath gas flow rate of 50, auxiliary gas flow rate of 20, sweep gas flow rate of 20 (arbitrary units for all gas flow rates), auxiliary gas heater temperature of 400 °C, ion transfer capillary temperature of 300 °C, spray voltage of 2.8 kV in negative mode and 3.5 kV in positive mode, and S-lens radio frequency of 50. The MS1 scan parameters were set to: AGC, 1 × 10^6^; maximum injection time, 250 ms; micro-scans, 1; resolution, 30,000; scan range, 75–1000 *m*/*z*. The MS2 scan parameters were set to: AGC, 1 × 10^5^; maximum injection time, 50 ms; isolation window, 4 *m*/*z*; collision energy, 30 V; resolution, 30,000; loop count, 5; dynamic exclusion time, 7 s. All metabolites for HILIC measurements were identified at confidence level 1 [62]. Peak areas were integrated and exported to Microsoft Excel via the Thermo TraceFinder software (version 5.1). The HILIC data (non-SIL and SIL) were normalized and analyzed as described for the ICMS data.

## MitoSOX measurements

HAP1 cells (3.75 × 10^5^) were seeded in 35-mm glass-bottom dishes in 2 mL of high-glucose DMEM medium (DMEM with 25 mM glucose and supplemented with 10% FBS and 1% penicillin/streptomycin) and allowed to attach overnight in a humidified incubator at 37 °C and 5% CO_2_. The next day, cells were exposed to galactose- or glucose-containing media (DMEM5030 with 10% FBS, 4 mM glutamine, 25 mM galactose or glucose, 100 units/mL penicillin, and 100 µg/mL streptomycin) to induce metabolic stress (with galactose) for 72 h. On the day of imaging, cells were stained with the live cell imaging dye MitoSOX Red (M36008, ThermoFisher) as per the manufacturer’s protocol. A total of 15 images were captured per dish at an excitation wavelength of 488 nm using a Dragonfly spinning disk confocal microscope at 40× magnification, and all images for an experiment were captured using the same microscope intensity settings. Phase contrast images were captured for some experiments at the same time to calibrate and measure cell size. Relative fluorescence intensity was measured at 525 nm by using a custom CellProfiler protocol with custom modules [64]. The image metadata were used to set the intensity range of the fluorescent image when importing images with the NamesAndTypes module. Images were imported and cells were classified as the primary objects using IdentifyPrimaryObjects module. The object size was calibrated on the basis of the size measurement of cells from the phase contrast images. The relative fluorescence intensity of the object from the fluorescent image was measured using MeasureObjectIntensity module. Raw data were exported using the ExportToSpreadsheet module. The integrated intensity (arbitrary intensity units), which is the sum of the pixel intensities within the object, was analyzed.

## Mitochondrial morphometric analysis

HAP1 cells were plated in a Cell Carrier-384-Ultra microscopy plate at 5000 cells/well or 7500 cells/well in a total volume of 50 µL. After 24 h incubation in IMDM, the medium was changed to either glucose- or galactose-containing basal medium (with or without supplementation of 2 mM nicotinic acid or nicotinamide), as described above. Triple stain 2× solution was prepared in cell culture medium, using Hoechst 33,342 at 2 µg/mL (ThermoFisher Scientific), MitoTracker Green FM (ThermoFisher Scientific) at 200 nM, and tetramethylrhodamine, methyl ester (TMRM, ThermoFisher Scientific) at 20 nM. After 48 h cell exposure to the different culture conditions, 25 µL of medium were removed and replaced with 25 µL of triple stain 2× solution using the Biomek FX^p^ Laboratory Automation Workstation (Beckman Coulter Life Sciences). Both media aspiration and triple stain 2× solution dispensing were performed with the end of the tips following the well position at 1 mm below liquid surface, with a flow of 5 µL/s. This procedure minimized cell disturbances due to mechanical forces or strong flow, while avoiding bubble formation. The chemical compounds FCCP and oligomycin were then transferred using the ECHO 550 Acoustic Liquid handler (Beckman Coulter Life Sciences) by adding 50 nL/well of 10 mM stock solutions in dimethyl sulfoxide to reach final concentrations of 10 µM and 0.1% of dye and DMSO respectively. After 45 min of incubation at 37 °C in the dark, cultures were washed three times with cell culture media containing TMRM (10 nM). Each combination of conditions was represented at least in 12 wells as technical replicates. Images were acquired using the CellVoyager CV8000 High-Content Screening System (Yokogawa) with the following settings: lasers 405 nm, 488 nm, and 561 nm at 100% power, acquisition using 60 × W objective (BP445/45 200 ms, BP525/50 250 ms, BP600/37 250 ms) without binning, 7 random fields per well. Hoechst acquisition was performed first, followed by simultaneous acquisition of Mitotracker Green and TMRM to minimize crosstalk between channels. Image analysis was performed using the CellPathfinder High Content Analysis Software (Yokogawa).

## Seahorse analysis

Oxygen consumption rate (OCR) was measured using the Seahorse XF Cell Mito Stress assay (Agilent XFe96), following the manufacturer’s recommendations. The modulators of mitochondrial function [oligomycin, carbonyl cyanide-4 (trifluoromethoxy) phenylhydrazone (FCCP), rotenone, and antimycin A] were added at different time points as per the manufacturer’s recommendations. IMDM-cultured HAP1 cells were seeded at a density of 2.5 × 10^4^ cells per well and received 2 µM oligomycin and 0.25 µM FCCP. DMEM-cultured HAP1 cells were seeded at a density of 1.5 × 10^4^ cells per well and received 1 µM oligomycin and 0.5 µM FCCP. Both the IMDM- and DMEM-cultured cells received 0.5 µM of rotenone and antimycin A. Twenty-four hours after seeding, media were replaced by DMEM, and another 24 h later, OCR measurements were made. Normalization was performed to the amount of DNA per well, which was determined using Cyquant (C7026, ThermoFisher Scientific). The data were processed with the Wave 2.6.1 software (Agilent).

## Expression and purification of human PHGDH and PSAT

Bacterial expression plasmids pET28a (IPTG-inducible T7 promoter, N-terminal His_6_ tag) containing the coding sequence of human PHGDH (hPHGDH) and pET15b (IPTG-inducible T7 promoter, N-terminal His_6_ tag) containing the coding sequence of human PSAT1 (encoding phosphoserine aminotransferase, the second enzyme in the serine biosynthesis pathway) were kind gifts from G. Bommer and E. Van Schaftingen [29]. Both recombinant proteins were expressed in *E. coli* BL21(DE3) cells transformed with the respective plasmids. The cells were grown in LB broth medium at 37 °C with continuous shaking until OD_600 nm_ reached 0.6–0.8, at which point a cold shock was applied to the cells for 20 min. This treatment was followed by induction of protein expression by IPTG (1 mM) and subsequent overnight shaking at 18 °C. After harvesting the cells by centrifugation, the cell pellets were resuspended in 25 mL of extraction buffer per liter of culture, containing 50 mM Tris (pH 7.5), 500 mM NaCl, 20 mM imidazole,1 U of DNAse (Invitrogen), and half a pill of an EDTA-free anti-protease cocktail (cOmplete tablets, EDTA-free protease inhibitor cocktail, Roche). Cells were lysed by sonication on ice (21 cycles of 5-s pulses followed by 60-s cooling intervals between the pulses and 50% amplitude). The crude extract was then centrifuged for 30 min at 17,000*g* and 4 °C, and the resulting supernatant loaded onto a nickel-affinity column using an ÄKTA protein purifier system (GE Healthcare). After washing with a buffer containing 50 mM Tris (pH 7.5), 20 mM imidazole, and 500 mM NaCl, the bound protein was eluted with an imidazole gradient (0–250 mM over 20 min with a flow rate of 1 mL/min) in this buffer. The affinity purification was followed by gel filtration on a Superdex 200 10/30 GL column (GE Healthcare) at a flow rate of 0.75 mL/min, carried out on the ÄKTA protein purifier system. The buffer used for this step was composed of 25 mM Tris (pH 7.5) and 150 mM NaCl. The concentration of the purified protein was estimated by measuring *A*_280 nm_ and using an extinction coefficient estimated by ProtParam (RRID: SCR_018087; https://web.expasy.org/protparam/). The protein purity for both purified enzymes (PHGDH and PSAT1) was estimated at > 95% by SDS–polyacrylamide gel electrophoretic analysis. The purified proteins were stored at −80 °C after addition of 10% glycerol.

## Western blot analysis

PHGDH levels were evaluated in HAP1 cells and patient-derived fibroblasts by Western blot analysis. To obtain total soluble proteins, cells were scraped from six-well plates (as described previously) using RIPA Lysis Buffer 1× (20–188, EMD Millipore) containing an EDTA-free anti-protease cocktail (cOmplete Tablets, Roche). Protein estimation was done using the bicinchoninic acid (BCA) assay kit (23,225, Pierce BCA Protein Assay Kit, Thermo Scientific). Human recombinant PHGDH (10 ng) and cellular extracts (10 µg) were loaded and separated by SDS–polyacrylamide gel electrophoresis (XP04200BOX, Novex 4–20%, Tris–Glycine Plus WedgeWell Gel, Thermo Scientific).

Proteins were transferred onto a nitrocellulose membrane (LC2009, Thermo Scientific) via dry transfer. Proteins were detected using anti-human PHGDH (Sigma-Aldrich, cat. no. HPA021241, RRID: AB_1855299, 1:500) and anti-human β-actin (Sigma-Aldrich, cat. no. A3854, RRID: AB_262011, 1:10,000) antibodies in Tris-buffered saline and 0.1% Tween-20 with 3% BSA. As secondary antibodies, goat anti-rabbit IgG (IRDye 800CW, LI-COR, cat. no. 926–32,211, RRID: AB_621843, 1:5000) and goat anti-mouse (IRDye 680RD, LI-COR, cat. no. 926–68,070, RRID: AB_10956588, 1:5000) were diluted in Intercept (PBS) Blocking Buffer (927–70,001, LI-COR). Fluorescence detection was performed in the LI-COR Odyssey FC (LI-COR 2800FC, serial no. OFC-0794) and processed using Image Studio software (LI-COR, version 5.2). Protein band intensities were estimated with ImageJ software (NIH, version 1.54 g).

## Enzymatic assays

The dehydrogenase activity of hPHGDH was determined at 30 °C, and the reaction was started with addition of the hPHGDH enzyme. The assay mixture contained 25 mM Tris (pH 9), 1 mM dithiothreitol, 1 mM MgCl_2_, 5 µM pyridoxal phosphate, 1 mM L-glutamate, 400 mM KCl, 0.5 mM NAD^+^, 100 µg/mL PSAT, and 6 µg/mL hPHGDH, in addition to the substrate 3-phosphoglycerate (3-PG), as described previously [29]. Initial velocities were measured at subsaturating (40 µM) or saturating (300 µM) concentrations of 3-PG. The formation of NADH was followed spectrophotometrically at 340 nm. Initial velocities were determined using the molar absorptivity of NADH (6220 M^−1^ cm^−1^). For the inhibition assays, purified *S*-, *R*-, or cyclic NADHX forms (0–150 µM) were added separately into the reaction mixture at indicated concentrations, and reaction velocities were measured as described above. For measurement of PHGDH activity in HAP1 cell extracts, an identical assay protocol was followed except that the extract was added to start the reaction (instead of purified hPHGDH). For preparation of extracts, HAP1 cells were grown in a basal medium containing glucose or galactose for 72 h in standard 10-cm Petri dishes. The cells were scraped after addition of 400 µL lysis buffer (50 mM HEPES pH 7.1 with 1× EDTA-free anti-protease inhibitor cocktail from Roche), and the crude extracts were centrifuged at 17,000*g* for 30 min at 4 °C. The supernatant was then loaded onto a 1-mL HiTrap Q HP column (29,051,325, Cytiva), which had been previously equilibrated with buffer A (25 mM HEPES, pH 7.1) at a flow rate of 0.5 mL/min. The column was washed with buffer A. Elution was performed with a linear salt gradient (0–1000 mM NaCl) over 20 min using buffer B (25 mM HEPES, pH 7.1, 2 M NaCl). Fractions of 1 mL were collected, and the peak fractions were used for the spectrophotometric assay of PHGDH activity.

## Generation of HAP1 rescue lines

The recombinant plasmids pLX301-CytoNAXD and pLX301-MitoNAXD, adapted for expression in human cells and used for subcellular compartment-specific rescue of NAXD expression in HAP1 cells knocked out for the *NAXD* gene, were generated by the means of the Gateway Technology according to the manufacturer’s instructions and as described previously [11]. In short, the pLX301-based expression plasmids containing the coding sequences for either eGFP (for generation of the transduction control lines Control_eGFP and NAXDko_eGFP) or the cytosolic or mitochondrial isoforms of human NAXD (for generation of the CytoNAXD and MitoNAXD rescue lines, respectively) were used for the generation of lentiviral vectors. Both the isoforms were cloned from pcDNA3.1 that contained the human full-length NAXD cDNA (RefSeq ID: NM_001242882.1; GenScript clone ID OHu08429) [11]. pLX301 was a gift from David Root (Addgene plasmid # 25,895) [65]. The lentiviral particles were generated in human embryonic kidney (HEK) 293 T cells via co-transfection of three plasmids (recombinant pLX301, containing the eGFP, CytoNAXD or MitoNAXD inserts, pCMV-δ8.2, and pCMV-VSVg) using Lipofectamine™ 2000 transfection reagent (Invitrogen), as detailed previously [11, 66]. Viral particles were harvested 72 h post-transfection from the spent culture medium, after filtration through a 0.45-µm membrane and concentration via PEG10,000 (sterile filtered)-mediated precipitation.

The HAP1 control and NAXDko cells were seeded at 3 × 10^5^ cells/well in a six-well plate, 24 h prior to the transductions with the respective collected viral particles. Puromycin (0.5 µg/mL cultivation medium) selection was started 48 h post-transduction, and as negative controls, non-transduced cells (to check their sensitivity to puromycin) as well as no puromycin-treated cells (to check their growth) were included. The transduced cells were kept on puromycin selection for 12 days, and then expanded and frozen down as DMSO stocks for further experiments.

## qPCR analysis

HAP1 cells (Control_eGFP, NAXDko_eGFP, CytoNAXD, and MitoNAXD lines) were grown in IMDM medium and collected using trypsinization. The cells were washed with PBS and centrifuged, and the pellets were plunged in liquid N_2_ and stored at −80 °C until analysis. RNA extraction was performed with the Qiagen RNeasy kit, following the manufacturer’s protocol, and with a homogenization step by QIAshredder (Qiagen) prior to extraction. The extracted RNA (2 µg; estimated using NanoDrop, ThermoFisher Scientific) from each cell line was used to synthesize cDNA using the SuperScript III Reverse Transcriptase kit (ThermoFisher Scientific), following the manufacturer’s protocol. Quantitative PCR (qPCR) reactions were performed in a 384-well plate in a 10 µL reaction containing 5 µL iQ SYBR Green Supermix (Bio-Rad), 0.3 µL forward primer (10 µM, final concentration 300 nM), 0.3 µL reverse primer (10 µM, final concentration 300 nM), 3.4 µL Milli-Q water, and 1 µL cDNA. The qPCR reactions were run in a thermocycler (Biometra Analytik TProfessional) with the following reaction conditions: initial denaturation at 95 °C for 10 min followed by 35 cycles of denaturation (95 °C, 30 s), annealing (60 °C, 30 s), and elongation (72 °C, 30 s). The primers used in the study (see also Supplementary Fig. S11A) were: NAXD_1 (forward), GGGCTTGCAGACGAGTTTTAG and NAXD_2 (reverse), TCCATCTTGCCCTTTGTGCT, for amplification of the Mitosignal; NAXD_5 (forward), AAACAAATGGGTCCAGCCCT and NAXD_6 (reverse), CTTCTGGAAGGCTTGGTGGT, for amplification of the Cytosignal; ACTB (forward) TGACCCAGATCATGTTTGAGACC and ACTB (reverse) AGGGATAGCACAGCCTGGAT, for amplification of the housekeeping gene used as a reference. The fold changes in expression were calculated by the Pfaffl method [67].

## Whole brain organoid derivations

A NAXDko iPSC line was derived from healthy male fibroblasts (GM03468; Coriell Institute; RRID: CVCL_7389) by the Gene Editing Core facility of the Murdoch Children’s Research Institute, Australia, using CRISPR/Cas9 technology. As will be described in detail in a separate article, to delete the *NAXD* gene, two Cas9 sites (ACAACGACCGCGCCGGCGA and GGCTGCATGCTCTTGTCGT) were targeted within exon 1 and exon 5. NAXDko iPSC clones were karyotyped by single-nucleotide polymorphism (SNP) array, sequence verified, confirmed to be mycoplasma negative, and verified for expression of pluripotency markers. Whole brain organoids (WBOs) were derived from NAXDko iPSCs and isogenic control iPSCs (Fig. 8D) according to the protocol described by Lancaster and Knoblich [40]. The organoids were grown in 24-well plates (1 organoid per well) in an orbital shaker incubator (37 °C, 5% CO_2_, 80 rpm).

For metabolomic experiments, the organoids were cultivated for 75 days in a rich medium [containing 25 mL DMEM-F12, 25 mL Neurobasal medium, 312.5 µL glutamine (200 mM), 250 µL N2 supplements, 12.5 µL insulin, 500 µL GlutaMAX, 250 µL MEM-non essential amino acids mix, 250 µL penicillin (10,000 units/mL), 250 µL streptomycin (10,000 µg/mL), 50 µL 2-mercaptoethanol (50 mM), 500 µL B27 with vitamin A] and shifted to a galactose-enriched medium [containing 25 mL DMEM-F12, 25 mL Neurobasal A without glucose/pyruvate, 312.5 µL glutamine (200 mM), 250 µL N2 supplements, 12.5 µL insulin, 500 µL GlutaMAX, 250 µL MEM-non essential amino acids mix, 250 µL penicillin (10,000 units/mL), 250 µL streptomycin (10,000 µg/mL), 50 µL 2-mercaptoethanol (50 mM), 500 µL B27 with vitamin A, and 12.5 mM galactose] for 72 h before metabolomic measurements. It should be noted that it was not possible to avoid glucose coming from DMEM-F12 medium in galactose-enriched medium.

## Whole brain organoid size, spent medium composition, and immunostaining

For size monitoring of WBOs during culture, areas were automatically determined using a Microplate Cytation5M Cell imaging Multi Mode Reader (Biotek).

The spent medium was collected, and absolute values for glucose, glutamine, and lactate concentrations in the spent medium were acquired using a YSI 2950D Biochemistry Analyzer (Kreienbaum KWM). All medium samples were filtered (Phenex-RC syringe filter, 0.2 µm; Phenomenex) prior to metabolite measurements. The instrument was calibrated and prepared according to the manufacturer’s instructions. For a precise and reliable quantification, external calibration curves of each analyte were prepared in the appropriate matrix and measured in duplicates.

Prior to performing immunostainings, WBOs were fixed with 4% paraformaldehyde overnight at 4˚C with shaking and then washed the next day three times with PBS for 15 min. Next, WBOs were embedded in 3% low-melting-point agarose in PBS. The solid agarose block was sectioned with a vibratome (Leica VT1000s) into 50-μm sections. For the immunostainings, sections were blocked for 90 min at room temperature on a shaker using 0.5% Triton X-100, 0.1% sodium azide, 0.1% sodium citrate, 5% normal goat serum, and 2% bovine serum albumin in PBS. Primary antibodies were diluted in the same solution and incubated with the sections for 48 h at 4 °C. Human SOX2 (R&D, cat no. AF2018, RRID: AB_355110, 1:100) and PAX6 (DSHB, RRID: AB_528427, 1:200) were used for neural stem cell evaluation. GFAP (Millipore, cat.no. AB5541, RRID: AB_177521, 1:1000) and S100β (Sigma, cat no. S2532, RRID: AB_477499, 1:600) were used for astrocyte assessment. Olig1 (Millipore, cat no. MAB344, RRID: AB_94860, 1:200) and Olig2 (Millipore, cat no. AB9610, RRID: AB_570666, 1:400) antibodies were used to evaluate the presence of oligodendrocyte progenitors in the WBOs. MAP2 antibodies (Abcam, cat no. ab32454, RRID: AB_776174, 1:100 and cat no. ab5392, RRID: AB_2138153, 1:1000) were used for neuronal characterization. After incubation with the primary antibodies, three 15-min washes with PBS were performed at room temperature. Subsequently, sections were incubated with secondary antibodies diluted in the same solution as the primary antibodies. All secondary antibodies (Invitrogen) were conjugated to Alexa Fluor fluorochromes and used in a 1:1000 dilution together with a Hoechst 33,342 (Life Technologies, cat no. H21492) nuclei counterstain. Secondary antibody incubations were performed for 2 h at room temperature with shaking and protected from light. Sections were then washed thrice with 0.05% Tween-20 in PBS for 5 min at room temperature and once with Milli-Q water and mounted with Fluoromount-G mounting medium (Southern Biotech, cat no. 0100–01) on a glass slide. Sections were dried overnight at room temperature and in the dark before imaging using confocal microscopy (LSM 710, Zeiss).

## Statistical analysis

An equal variance, unpaired Student’s *t*-test or ANOVA, as indicated, was applied to experimental data to determine statistically significant differences (*p < 0.05; **p < 0.01; ***p < 0.001; ns, not significant).

## Supplementary Information

Additional file 1.

Additional file 2.

Additional file 3.

Additional file 4.

## Data Availability

The datasets supporting the conclusions of this article are included within the article and its additional files.
