# Supplementary material for: Failure to repair damaged NAD(P)H blocks de novo serine synthesis in human cells
Source: Cell Mol Biol Lett. 2025 Jan 9;30:3. doi: 10.1186/s11658-024-00681-8 (PMC11715087; doi:10.1186/s11658-024-00681-8)
Supplement: Supplementary file 3 — Additional file 3. [file 11658_2024_681_MOESM3_ESM.zip › Supplementary Figures/FigureS11_for_fig5_revised.pdf]

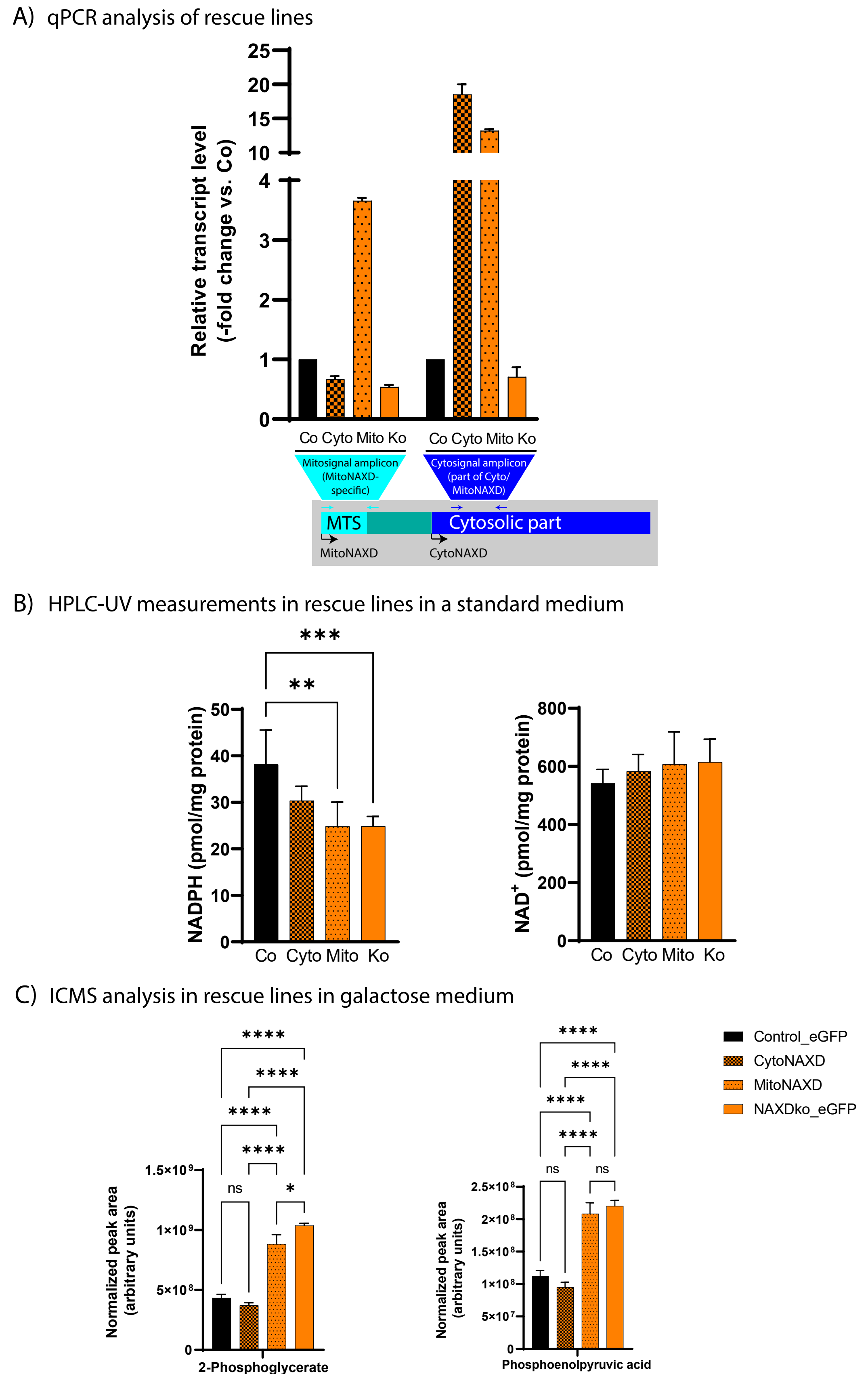
A) qPCR analysis of rescue lines
Relative transcript level
(-fold change vs. Co)
25
20
15
10
4
3
2
1
0
Co Cyto Mito Ko
Co Cyto Mito Ko
Mitosignal amplicon
(MitoNAXD-specific)
Cytosignal amplicon
(part of Cyto/ MitoNAXD)
MTS
Cytosolic part
MitoNAXD
CytoNAXD
B) HPLC-UV measurements in rescue lines in a standard medium
NADPH (pmol/mg protein)
50
40
30
20
10
0
***
**
Co Cyto Mito Ko
NAD+ (pmol/mg protein)
800
600
400
200
0
Co Cyto Mito Ko
C) ICMS analysis in rescue lines in galactose medium
Normalized peak area
(arbitrary units)
1.5×10⁹
1×10⁹
5×10⁸
0
****
ns
*
2-Phosphoglycerate
Normalized peak area
(arbitrary units)
2.5×10⁸
2×10⁸
1.5×10⁸
1×10⁸
5×10⁷
0
****
ns
Phosphoenolpyruvic acid
Control_eGFP
CytoNAXD
MitoNAXD
NAXDko_eGFP
