# Supplementary material for: Failure to repair damaged NAD(P)H blocks de novo serine synthesis in human cells
Source: Cell Mol Biol Lett. 2025 Jan 9;30:3. doi: 10.1186/s11658-024-00681-8 (PMC11715087; doi:10.1186/s11658-024-00681-8)

A) Serine supplementation does not rescue growth defect in NAXDko cells

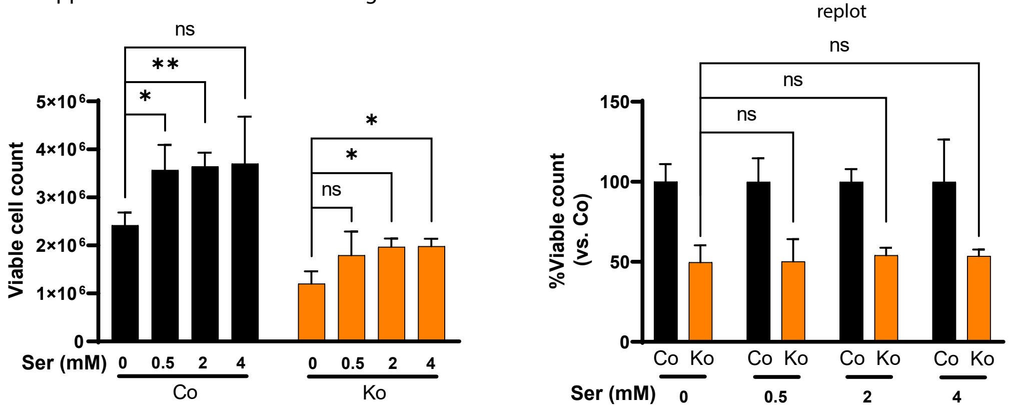

B) Inosine supplementation shows partial rescue of the growth defect in NAXDko cells

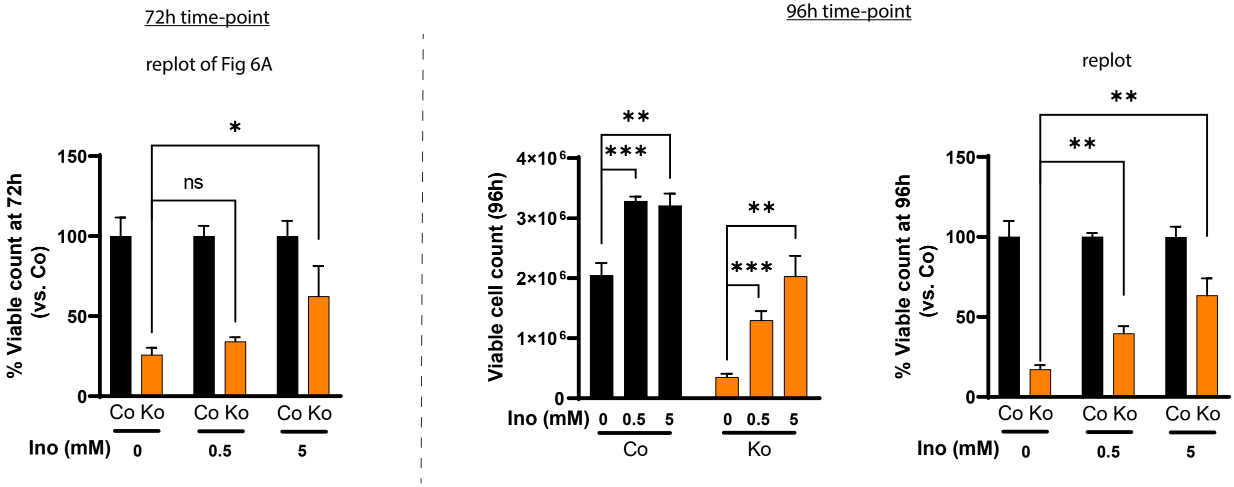

C) HPLC-UV measurements upon inosine supplementation

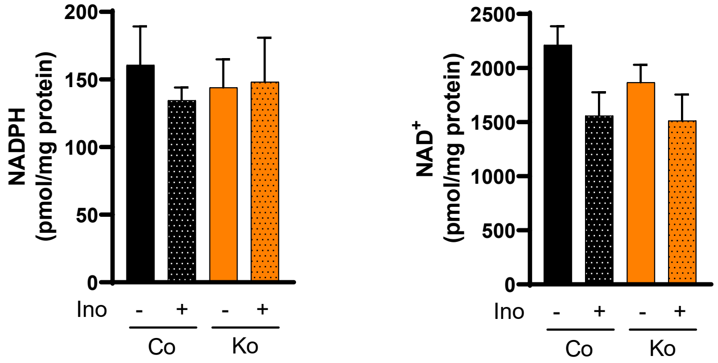

Supplement: Supplementary file 3 — Additional file 3. [file 11658_2024_681_MOESM3_ESM.zip › Supplementary Figures/FigureS12_for_fig6.pdf]
