# Supplementary material for: Failure to repair damaged NAD(P)H blocks de novo serine synthesis in human cells
Source: Cell Mol Biol Lett. 2025 Jan 9;30:3. doi: 10.1186/s11658-024-00681-8 (PMC11715087; doi:10.1186/s11658-024-00681-8)
Supplement: Supplementary file 3 — Additional file 3. [file 11658_2024_681_MOESM3_ESM.zip › Supplementary Figures/FigureS14_for_fig7.pdf]

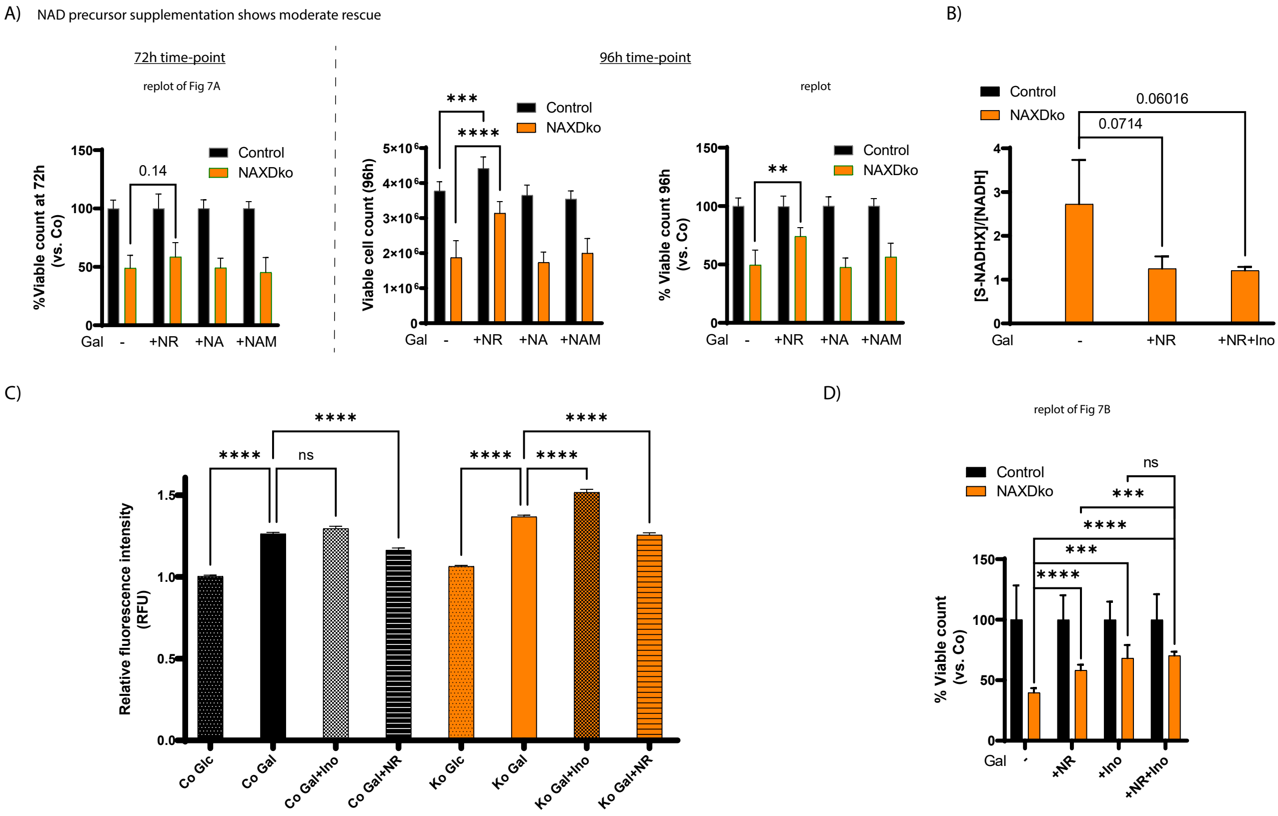

A) NAD precursor supplementation shows moderate rescue
72h time-point
replot of Fig 7A
%Viable count at 72h (vs. Co)
150
100
50
0
0.14
Control
NAXDko
Gal - +NR +NA +NAM
96h time-point
Viable cell count (96h)
5×10^6
4×10^6
3×10^6
2×10^6
1×10^6
0
***
****
Control
NAXDko
Gal - +NR +NA +NAM
replot
% Viable count 96h (vs. Co)
150
100
50
0
**
Control
NAXDko
Gal - +NR +NA +NAM
B)
Control
NAXDko
0.06016
0.0714
[S-NADHX]/[NADH]
4
3
2
1
0
Gal - +NR +NR+Ino
C)
Relative fluorescence intensity (RFU)
1.5
1.0
0.5
0.0
****
ns
****
****
****
****
Co Glc
Co Gal
Co Gal+Ino
Co Gal+NR
Ko Glc
Ko Gal
Ko Gal+Ino
Ko Gal+NR
D)
replot of Fig 7B
Control
NAXDko
ns
***
****
***
****
% Viable count (vs. Co)
150
100
50
0
Gal - +NR +Ino +NR+Ino
