# Supplementary material for: Failure to repair damaged NAD(P)H blocks de novo serine synthesis in human cells
Source: Cell Mol Biol Lett. 2025 Jan 9;30:3. doi: 10.1186/s11658-024-00681-8 (PMC11715087; doi:10.1186/s11658-024-00681-8)
Supplement: Supplementary file 3 — Additional file 3. [file 11658_2024_681_MOESM3_ESM.zip › Supplementary Figures/FigureS15_for_fig8.pdf]

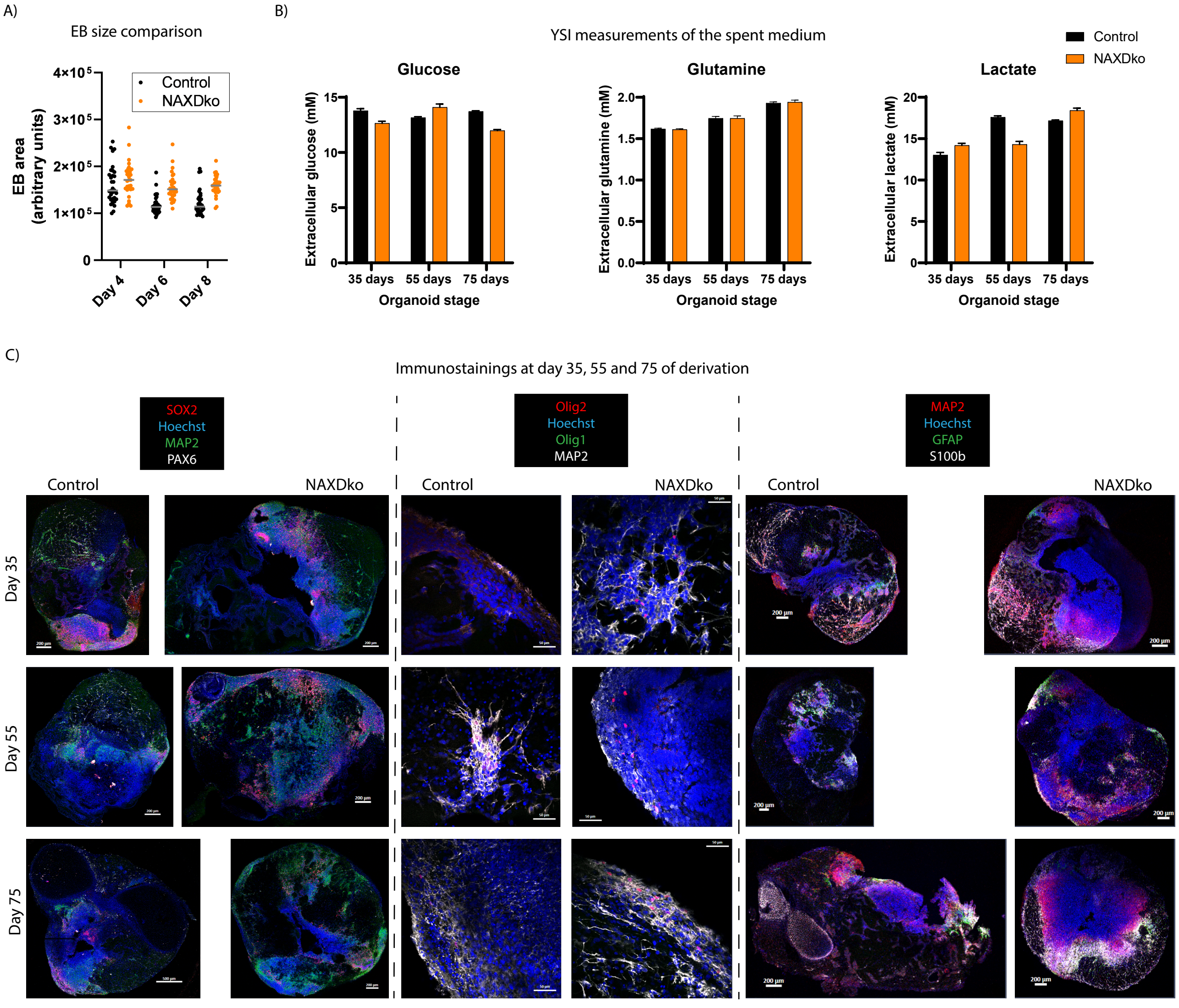
A)
EB size comparison
EB area (arbitrary units)
Control
NAXDko
Day 4
Day 6
Day 8
B)
YSI measurements of the spent medium
Control
NAXDko
Glucose
Extracellular glucose (mM)
Glutamine
Extracellular glutamine (mM)
Lactate
Extracellular lactate (mM)
35 days
55 days
75 days
Organoid stage
C)
Immunostainings at day 35, 55 and 75 of derivation
SOX2
Hoechst
MAP2
PAX6
Olig2
Hoechst
Olig1
MAP2
MAP2
Hoechst
GFAP
S100b
Control
NAXDko
Day 35
Day 55
Day 75
