# Supplementary material for: Failure to repair damaged NAD(P)H blocks de novo serine synthesis in human cells
Source: Cell Mol Biol Lett. 2025 Jan 9;30:3. doi: 10.1186/s11658-024-00681-8 (PMC11715087; doi:10.1186/s11658-024-00681-8)

A)

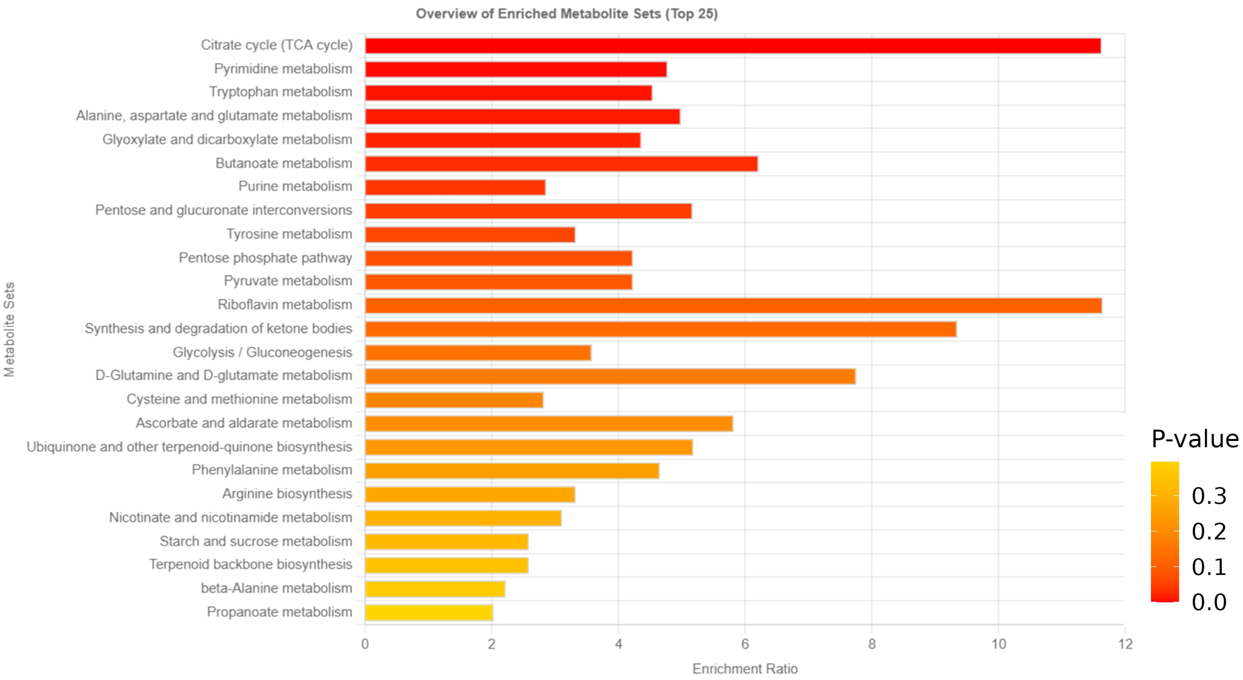

B)

	Metabolite Set	Total	Hits	Expect	P value	Holm P	FDR	Details
	Citrate cycle (TCA cycle)	20	5	0.43	4.13E-5	0.00347	0.00347	View
	Pyrimidine metabolism	39	4	0.838	0.00855	0.71	0.286	View
	Tryptophan metabolism	41	4	0.881	0.0102	0.838	0.286	View
	Alanine, aspartate and glutamate metabolism	28	3	0.602	0.0205	1.0	0.431	View
	Glyoxylate and dicarboxylate metabolism	32	3	0.688	0.0293	1.0	0.493	View
	Butanoate metabolism	15	2	0.322	0.0395	1.0	0.548	View
	Purine metabolism	65	4	1.4	0.0475	1.0	0.548	View

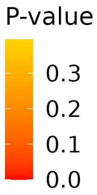

Supplement: Supplementary file 3 — Additional file 3. [file 11658_2024_681_MOESM3_ESM.zip › Supplementary Figures/FigureS2_for_fig1.pdf]
