# Supplementary material for: Failure to repair damaged NAD(P)H blocks de novo serine synthesis in human cells
Source: Cell Mol Biol Lett. 2025 Jan 9;30:3. doi: 10.1186/s11658-024-00681-8 (PMC11715087; doi:10.1186/s11658-024-00681-8)
Supplement: Supplementary file 3 — Additional file 3. [file 11658_2024_681_MOESM3_ESM.zip › Supplementary Figures/FigureS5_for_fig2.pdf]

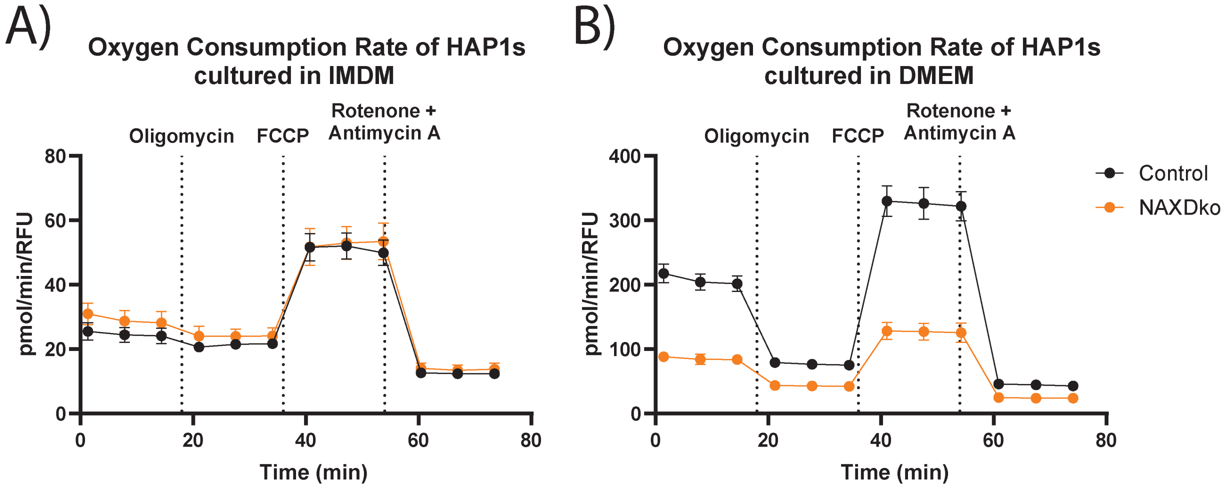
A)
Oxygen Consumption Rate of HAP1s cultured in IMDM
Oligomycin
FCCP
Rotenone + Antimycin A
pmol/min/RFU
0
20
40
60
80
Time (min)
B)
Oxygen Consumption Rate of HAP1s cultured in DMEM
Oligomycin
FCCP
Rotenone + Antimycin A
pmol/min/RFU
0
100
200
300
400
0
20
40
60
80
Time (min)
Control
NAXDko
