# Supplementary material for: Failure to repair damaged NAD(P)H blocks de novo serine synthesis in human cells
Source: Cell Mol Biol Lett. 2025 Jan 9;30:3. doi: 10.1186/s11658-024-00681-8 (PMC11715087; doi:10.1186/s11658-024-00681-8)

A) Determining seeding density for metabolite extractions in galactose medium

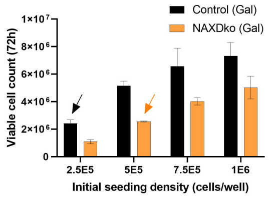

B) 3-Phosphoglycerate and 3-phosphoserine levels are not changed in NAXDko cells in glucose medium

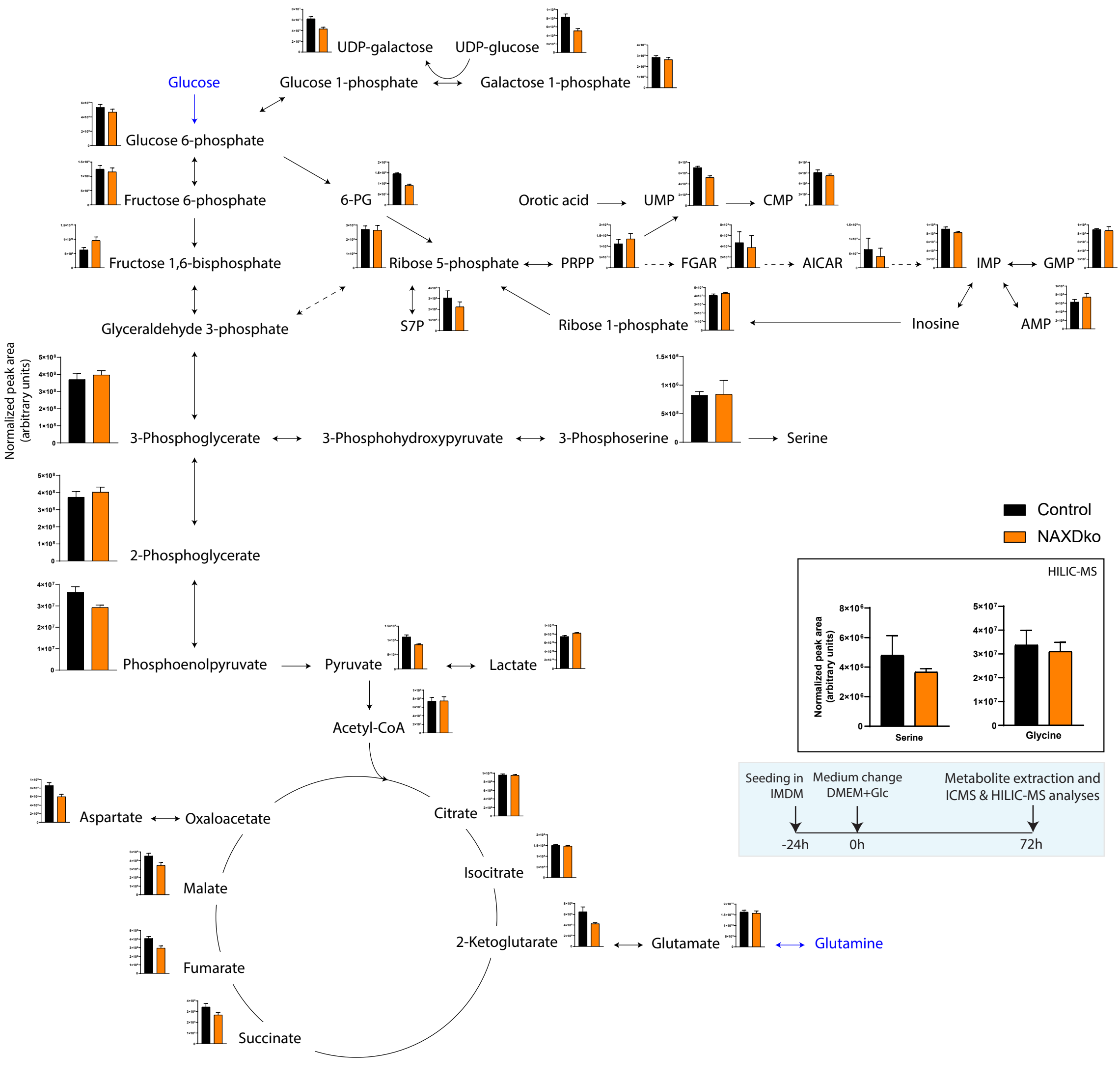

C) NAXEko cells do not show any signs of inhibition of serine biosynthesis

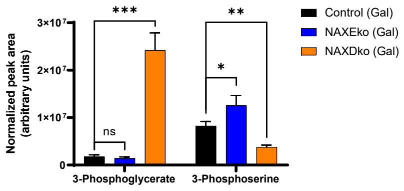

Supplement: Supplementary file 3 — Additional file 3. [file 11658_2024_681_MOESM3_ESM.zip › Supplementary Figures/FigureS7_for_fig3_revised.pdf]
