# Supplementary material for: Failure to repair damaged NAD(P)H blocks de novo serine synthesis in human cells
Source: Cell Mol Biol Lett. 2025 Jan 9;30:3. doi: 10.1186/s11658-024-00681-8 (PMC11715087; doi:10.1186/s11658-024-00681-8)
Supplement: Supplementary file 3 — Additional file 3. [file 11658_2024_681_MOESM3_ESM.zip › Supplementary Figures/FigureS9_for_fig4.pdf]

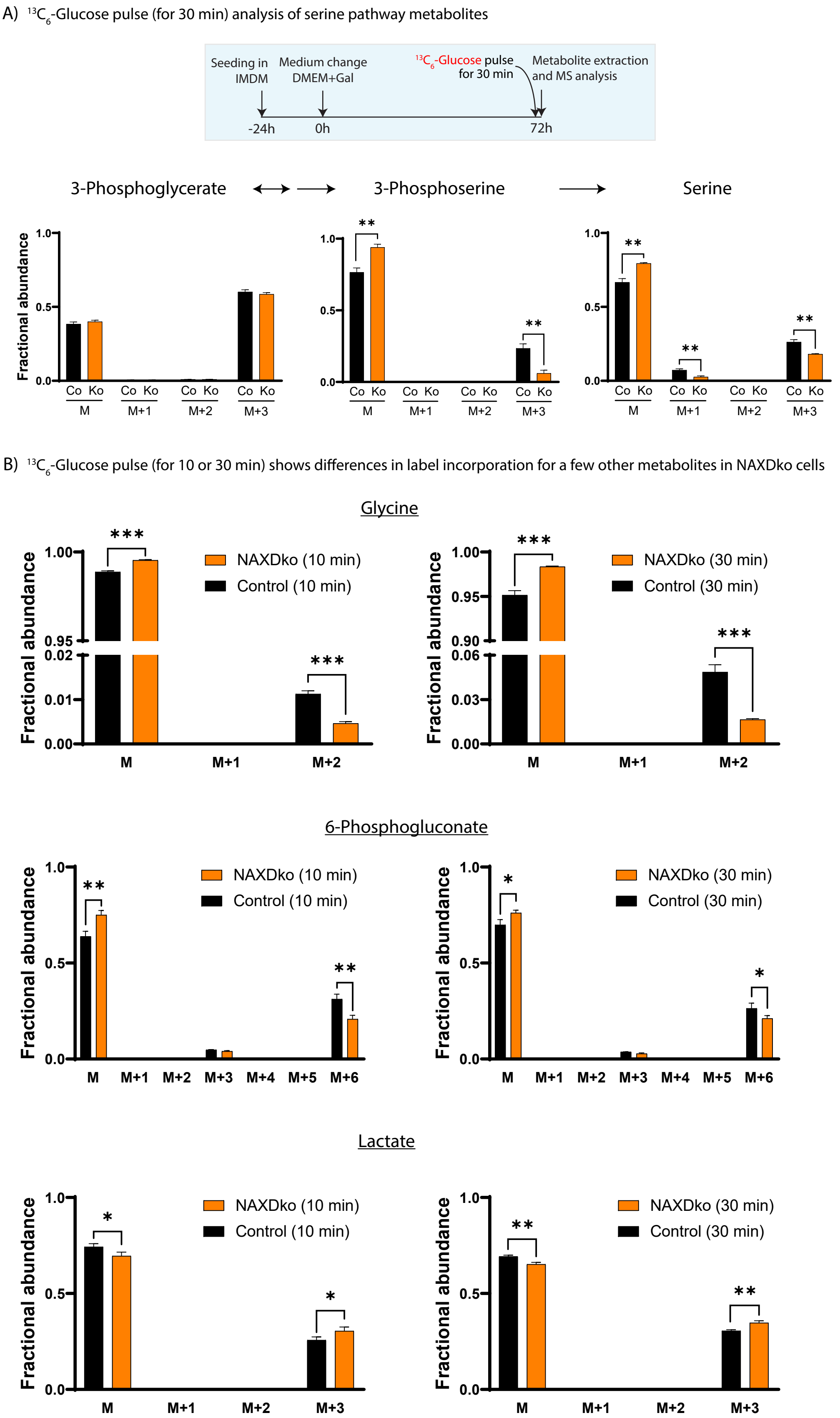

A) 13C6-Glucose pulse (for 30 min) analysis of serine pathway metabolites
Seeding in IMDM
Medium change DMEM+Gal
13C6-Glucose pulse for 30 min
Metabolite extraction and MS analysis
-24h
0h
72h
3-Phosphoglycerate
3-Phosphoserine
Serine
Fractional abundance
1.0
0.5
0.0
Co Ko
M
M+1
M+2
M+3
**
B) 13C6-Glucose pulse (for 10 or 30 min) shows differences in label incorporation for a few other metabolites in NAXDko cells
Glycine
NAXDko (10 min)
Control (10 min)
NAXDko (30 min)
Control (30 min)
***
1.00
0.95
0.90
0.06
0.03
0.02
0.01
0.00
6-Phosphogluconate
M+4
M+5
M+6
*
Lactate
