# Supplementary figures and images for: Failure to repair damaged NAD(P)H blocks de novo serine synthesis in human cells
Source: Cell Mol Biol Lett. 2025 Jan 9;30:3. doi: 10.1186/s11658-024-00681-8 (PMC11715087; doi:10.1186/s11658-024-00681-8)

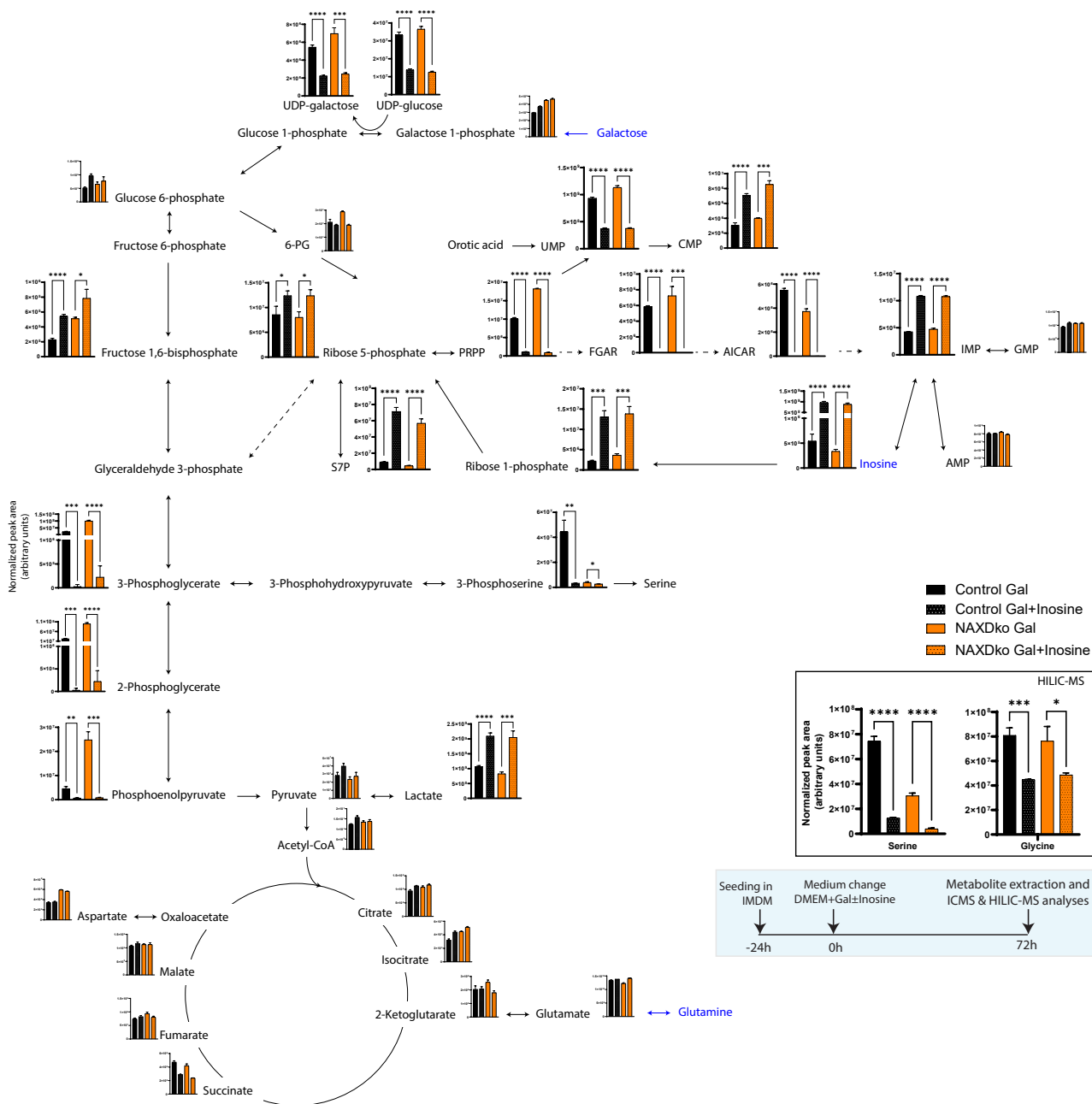

Supplement: Supplementary file 3 — Additional file 3. [file 11658_2024_681_MOESM3_ESM.zip › Supplementary Figures/FigureS13_for_fig6.pdf]

A)

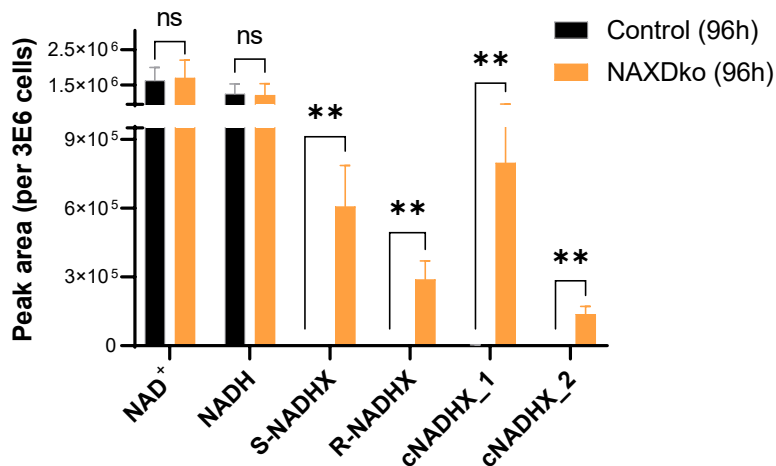

B) GC-MS analysis

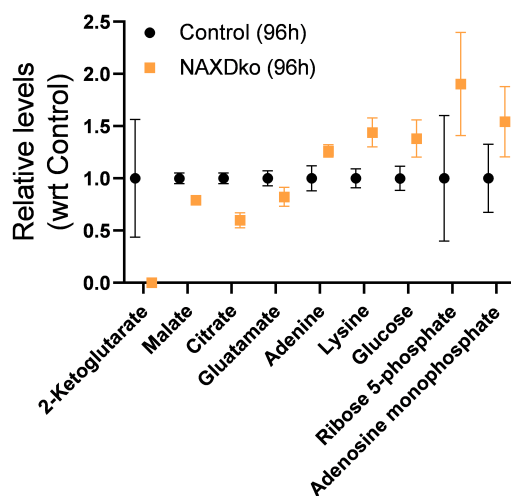

C) LC-MS analysis

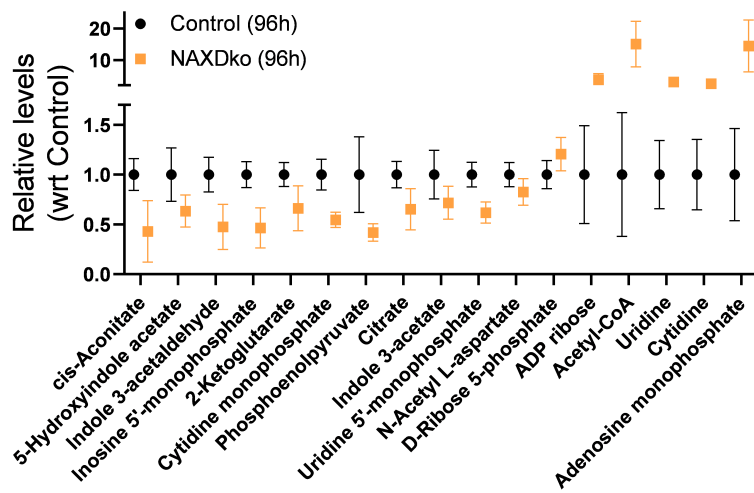

Supplement: Supplementary file 3 — Additional file 3. [file 11658_2024_681_MOESM3_ESM.zip › Supplementary Figures/FigureS1_for_fig1.pdf]

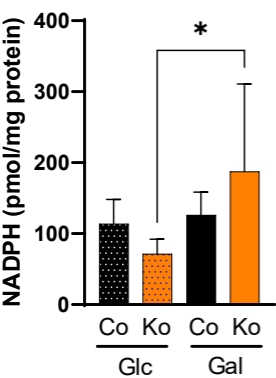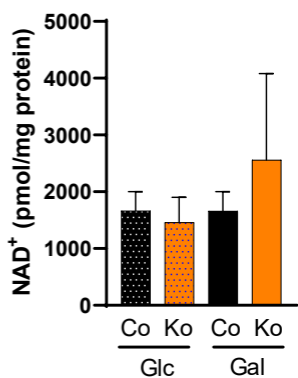

Supplement: Supplementary file 3 — Additional file 3. [file 11658_2024_681_MOESM3_ESM.zip › Supplementary Figures/FigureS3_for_fig2.pdf]

A)

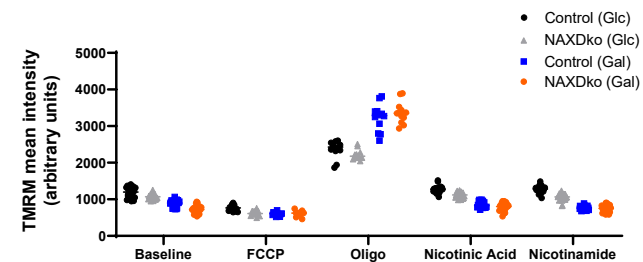

B)

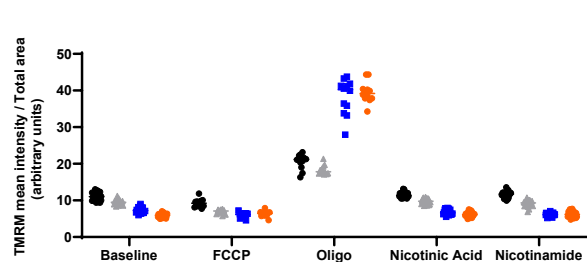

C)

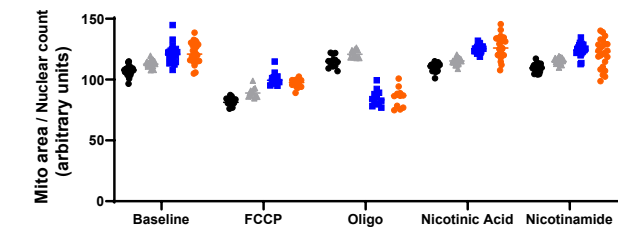

D)

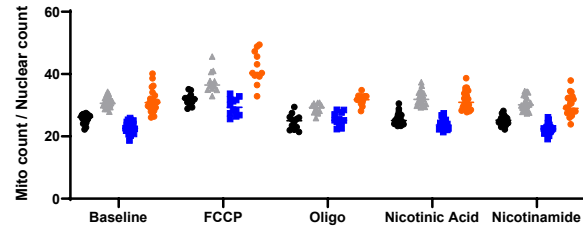

E)

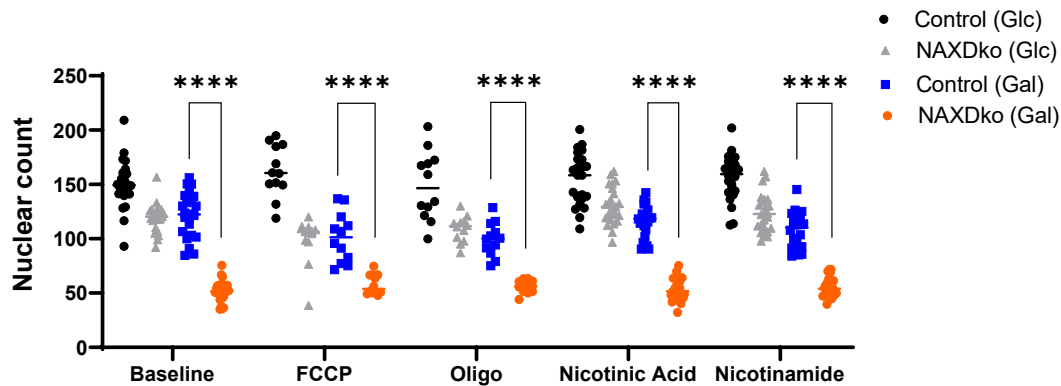

Supplement: Supplementary file 3 — Additional file 3. [file 11658_2024_681_MOESM3_ESM.zip › Supplementary Figures/FigureS4_for_fig2_revised.pdf]

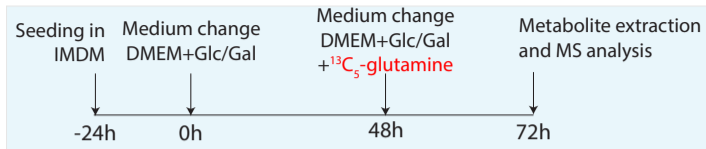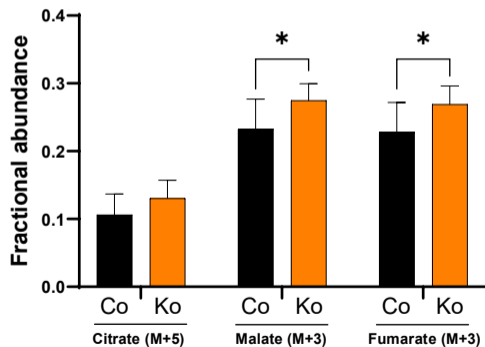

Glucose condition

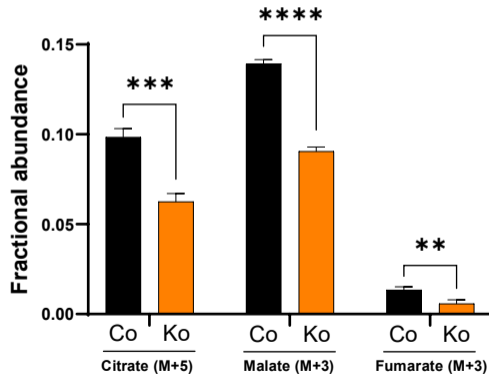

Galactose condition

Supplement: Supplementary file 3 — Additional file 3. [file 11658_2024_681_MOESM3_ESM.zip › Supplementary Figures/FigureS6_for_fig2.pdf]

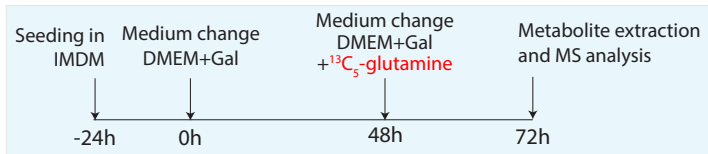

3-Phosphoglycerate

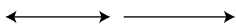

3-Phosphoserine

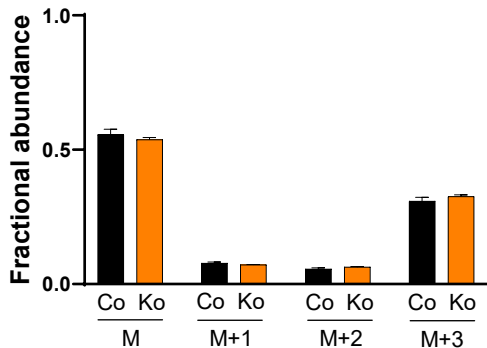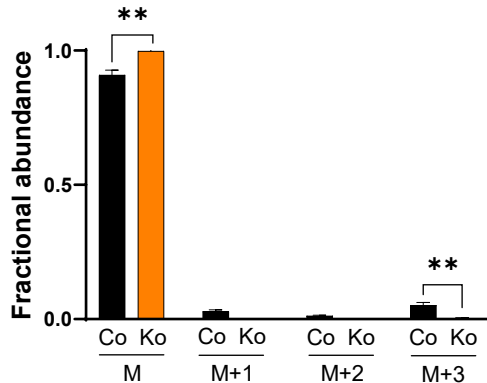

Supplement: Supplementary file 3 — Additional file 3. [file 11658_2024_681_MOESM3_ESM.zip › Supplementary Figures/FigureS8_for_fig4.pdf]
