# Supplementary material for: Failure to repair damaged NAD(P)H blocks de novo serine synthesis in human cells
Source: Cell Mol Biol Lett. 2025 Jan 9;30:3. doi: 10.1186/s11658-024-00681-8 (PMC11715087; doi:10.1186/s11658-024-00681-8)
Supplement: Supplementary file 4 — Additional file 4. [file 11658_2024_681_MOESM4_ESM.pdf]

Supplementary Figure Legends

Supplementary Figure S1: A) HAP1 NAXDko cells show accumulation of different forms of NADHX. Control and NAXDko cells were grown in IMDM for 72 h (Figure 1B) or 96 h (this panel), and metabolite extracts were analyzed by LC-MS. All values were normalized to an internal standard and to cell counts done in a replicate plate and are means ± SDs of three biological replicates. Statistical significance was calculated using an equal variance, unpaired Student's t-test (**$p < 0.01$; ns, not significant). Metabolite extracts from HAP1 control and NAXDko cells grown for 96 h in IMDM were analyzed by GC-MS (B) and by LC-MS (C). All values presented are relative to control cells and are means ± SDs of six biological replicates. Data generated from this experiment at the 72 h time point are shown in Figure 1C-D. Acetyl-CoA, acetyl coenzyme A; ADP, adenosine diphosphate.

Supplementary Figure S2: Metabolite enrichment analysis based on significantly changed and annotated metabolites between HAP1 control and NAXDko cells grown in IMDM for 72 h and 96 h (metabolites detected with LC-MS only). A) Top 25 enriched metabolite sets are presented along with their enrichment ratio. The color gradient indicates p-values. B) The most significantly enriched sets ($p < 0.05$) are presented along with the number of hits and total number of genes in the respective enriched sets.

Supplementary Figure S3: Control and NAXDko cells were grown for 72 h in glucose or galactose, and NAD(P)(H)(X) levels were measured by HPLC-UV. NADPH and NAD^+ levels are shown here; S-NADHX, R-NADHX, and NADH are shown in Figure 2B. All values are means ±

SDs of six biological replicates (except for the control galactose condition, where four replicates were analyzed). Statistical significance was calculated using an equal variance, unpaired Student's t-test (*$p < 0.05$). Co, Control; Ko, NAXDko; Glc, glucose medium; Gal, galactose medium.

Supplementary Figure S4: Morphometric analyses of HAP1 control and NAXDko cells cultivated in glucose or in galactose medium. The cells were seeded in IMDM and changed to basal medium with glucose or galactose, without or with supplementation of 2 mM nicotinic acid or nicotinamide. After 48 h of growth in the respective media, the cells were stained with Hoechst 33342, MitoTracker Green FM, and TMRM and treated with DMSO (as control), 40 µM FCCP or 10 µg/mL oligomycin, before morphometric analyses using high-content imaging. Panel A shows total TMRM mean intensity, panel B shows TMRM mean intensity normalized to total mitochondrial area, panel C shows total mitochondrial area, and panel D shows the ratio of mitochondrial to nuclear count. Panel E shows nuclear counts which only differed significantly between control and NAXD KO cells under the galactose culture conditions, without or with treatment with mitochondrial modulators or NAD precursors. Each dot represents an individual data point and median values are indicated by a horizontal line. Statistical significance was calculated using an equal variance, unpaired Student's t-test (****$p < 0.0001$). Oligo, oligomycin; Mito, mitochondrial; Glc, glucose medium; Gal, galactose medium.

Supplementary Figure S5: Seahorse analyses of HAP1 control and NAXDko cells in different media. The cells were seeded and grown for 24 h in either A) IMDM or B) DMEM, both

containing glucose as the carbon source. Media were then exchanged for fresh DMEM and, 24 h later, the oxygen consumption rate (OCR) of the cells was recorded at the basal state and after the successive additions of oligomycin, FCCP, and rotenone/antimycin A. Data shown are means ± SDs of at least five biological replicates.

Supplementary Figure S6: Testing reductive carboxylation of glutamine in HAP1 NAXD deficient cells. HAP1 control and NAXDko cells were grown in glucose or galactose medium as shown in the inset, and medium was changed to a tracer medium containing $^{13}C_5$-glutamine (keeping the rest of the components identical) to track the reductive carboxylation of the labeled glutamine. Increased reductive carboxylation is indicated by increased formation of M+5 citrate, M+3 malate and M+3 fumarate [32]. The NAXDko cells grown in glucose showed a slightly increased fractional abundance for the M+3 signals of malate and fumarate, whereas in galactose reduced label incorporation was measured for NAXDko cells for M+5 citrate, M+3 malate and M+3 fumarate. All values are means ± SDs of nine (glucose condition) and three (galactose condition) biological replicates. Statistical significance was calculated using an equal variance, unpaired Student's t-test (*$p < 0.05$; **$p < 0.01$; ***$p < 0.001$; ****$p < 0.0001$). Co, Control; Ko, NAXDKo; Gal, galactose; Glc, glucose; MS, mass spectrometry.

Supplementary Figure S7: A) Determining cell seeding density for metabolite extractions in galactose medium. Control and NAXDko cells were grown in galactose medium for 72 h, starting with the indicated initial seeding densities. All values are means ± SDs of three biological replicates. Similar final cell counts were reached after 72 h with initial seeding densities of 2.5×10^5 cells/well for control cells (black arrow) and of 5×10^5 cells/well for

NAXDko cells (orange arrow), and these were used for further metabolite profiling experiments in galactose medium. B) 3-Phosphoglycerate and 3-phosphoserine levels are not changed in NAXDko cells compared to control cells in glucose medium. Control and NAXDko cells were seeded in IMDM and shifted to glucose- or galactose-containing medium for 72 h before metabolite extraction and analysis by ICMS. The metabolite levels were normalized to summed MS1 intensities within each sample. All values are means ± SDs of three biological replicates. Statistical significance was calculated using an equal variance, unpaired Student's t-test (***$p < 0.001$; ****$p < 0.0001$; ns, not significant). C) HAP1 NAXEko cells do not show any signs of inhibition of serine biosynthesis. Control, NAXDko, and NAXEko cells were grown in galactose medium for 72 h before metabolite extraction and analysis by ICMS. The metabolite levels were normalized to summed MS1 intensities within each sample. All values are means ± SDs of three biological replicates. Statistical significance was calculated using an equal variance, unpaired Student's t-test (*$p < 0.05$; **$p < 0.01$, ***$p < 0.001$; ns, not significant). Glc, glucose medium; Gal, galactose medium.

Supplementary Figure S8: Tracer analysis with $^{13}C_5$-glutamine shows reduced label incorporation into 3-phosphoserine in NAXDko cells. The experimental scheme is shown in the inset. Control and NAXDko cells were grown in galactose for 48 h and then medium was changed to a tracer containing medium (keeping other components identical to the unlabeled medium). The cells were grown for another 24 h and then metabolites were extracted for ICMS analysis. Fractional distribution of the label is shown for 3-phosphoglycerate and 3-phosphoserine. All values are means ± SDs of three biological replicates. Statistical

significance was calculated using an equal variance, unpaired Student's t-test (***p* < 0.01). Co, Control; Ko, NAXDKo; Gal, galactose; MS, mass spectrometry.

Supplementary Figure S9: Tracer analysis with $^{13}C_6$-glucose for control and NAXDko cells in galactose medium. Control and NAXDko cells grown in galactose were pulsed with $^{13}C_6$-glucose for 10 min or 30 min, followed by metabolite extraction and analysis using ICMS and HILIC-MS. A) Labeling data for serine pathway metabolites after 30 min of $^{13}C_6$-glucose pulse (corresponding data for the 10 min labeling pulse is shown in Figure 4A). B) Labeling data for glycine, 6-phosphogluconate, and lactate for the 10 min and 30 min labeling pulses with $^{13}C_6$-glucose. All values are means ± SDs of three biological replicates. Statistical significance was calculated using an equal variance, unpaired Student's t-test (**p* < 0.05; ***p* < 0.01, ****p* < 0.001). Co, Control; Ko, NAXDKo; Gal, galactose; MS, mass spectrometry.

Supplementary Figure S10: A) The purified fraction of His-tagged recombinant human PHGDH (hPHGDH) was analyzed by SDS-polyacrylamide gel electrophoresis and staining with Coomassie blue. The molecular weight ladder is marked with the respective kDa values. B) Western blot analysis of control and NAXD-deficient HAP1 cells and fibroblasts grown in galactose medium. The expected molecular weight for recombinant His-hPHGDH, endogenous hPHGDH, and β-actin is 58.7 kDa, 56.6 kDa, and 41.7kDa, respectively. As a positive control, 10 ng of recombinant His-hPHGDH was loaded. In all other lanes, 10 µg of protein was loaded. The intensity ratio was calculated separately for each lane by comparing the band intensities of PHGDH and β-actin within the same lane. C) PHGDH activity in extracts from glucose- or galactose-grown cells is susceptible to S-NADHX inhibition. HAP1 control and

NAXDko cells were grown in glucose or galactose and PHGDH activity was assayed, in the presence of 300 µM 3-PG and without or with exogenous S-NADHX (75 µM), in extracts prepared from these cells (at a final concentration of 0.74–1.3 mg protein/mL in the assay). Co, Control; Ko, NAXDKo.

Supplementary Figure S11: A) qPCR analysis of HAP1 rescue lines. The primer positions used for detecting transcripts containing the mitochondrial targeting sequence (MTS) and the shared cytosolic domain of *NAXD*, using the Mitosignal and Cytosignal primer pairs, respectively, are indicated. RNA extracted from the indicated cell lines grown in a standard IMDM medium was used for qPCR analysis. *ACTB* was used as a reference gene. Values are means ± SDs of three technical replicates. B) The rescue lines were grown along with the control lines in a standard IMDM medium for 72 h and NAD(P)(H)(X) levels were measured by HPLC-UV. NADPH and NAD^+ levels are shown here and NADH, S-NADHX, and R-NADHX levels are shown in Figure 5B. Values are means ± SDs of six biological replicates (except for the MitoNAXD line, where five replicates were analyzed). C) The rescue lines were grown in galactose for 72 h along with the control lines and metabolite extracts were analyzed by ICMS. Data was normalized using the summed MS1 intensities of all metabolites within each sample. All values are means ± SDs of three biological replicates. The levels of 2-phosphoglycerate and phosphoenolpyruvate are shown here. The data for other metabolites is shown in Figure 5C. Statistical significance was calculated using ordinary one-way ANOVA (*$p < 0.05$; ****$p < 0.0001$; ns, not significant). Co, Control; Cyto, CytoNAXD rescue line; Mito, MitoNAXD rescue line; Ko, NAXDko.

Supplementary Figure S12: A) Serine supplementation does not rescue growth specifically in the NAXDko cells. HAP1 control and NAXDko cells were grown in galactose media for 72 h without or with supplementation of serine at the indicated concentrations. In the replot, viability of the NAXDko cells is shown relative to control cells treated with the same concentration of serine. B) Inosine supplementation partially rescues growth in NAXDko cells. HAP1 control and NAXDko cells were grown in galactose media without or with supplementation of inosine at the indicated concentrations. The cell count was measured after 72 h (Figure 6A) or 96 h (this panel). The replots for both time points are also shown in this panel. C) Control and NAXDko cells were grown in galactose without or with 5 mM inosine supplementation for 72 h, and NAD(P)(H)(X) levels were analyzed by HPLC-UV. The levels of NADPH and NAD^+ from these measurements are shown here and levels of NADH, S-NADHX, and R-NADHX are shown in Figure 6B. All values are means ± SDs of at least three biological replicates. Statistical significance was calculated using an equal variance, unpaired Student's t-test (*$p < 0.05$; **$p < 0.01$, ***$p < 0.001$; ns, not significant). Co, Control; Ko, NAXDKo; Ser, serine; Ino, inosine.

Supplementary Figure S13: Metabolic profiling of HAP1 control and NAXDko cells without or with inosine supplementation. Control and NAXDko cells were grown without or with 5 mM inosine supplementation for 72 h, and metabolite extracts were analyzed using ICMS and HILIC-MS (shown in inset). The nutrient sources are marked with blue font. The complete mapped data is presented here, whereas measurements selected metabolites are shown in Figure 6C. Data was normalized using the summed MS1 intensities of all metabolites within each sample. All values are means ± SDs of three biological replicates. Statistical significance

was calculated using an equal variance, unpaired Student's t-test (*$p < 0.05$; **$p < 0.01$; ***$p < 0.001$; ****$p < 0.0001$). Gal, galactose; 6-PG, 6-phosphogluconate; S7P, sedoheptulose 7-phosphate; PRPP, phosphoribosyl pyrophosphate; FGAR, phosphoribosyl-N-formylglycinamide; AICAR, 5-aminoimidazole-4-carboxamide ribonucleotide; AMP, adenosine monophosphate; GMP, guanosine monophosphate; IMP, inosine monophosphate; CMP, cytidine monophosphate; UMP, uridine monophosphate; UDP, uridine diphosphate; Acetyl-CoA, acetyl coenzyme A; ICMS, ion-exchange mass spectrometry; HILIC-MS, hydrophilic interaction liquid chromatography- mass spectrometry

Supplementary Figure S14: A) NAD precursor supplementation exerts moderate phenotypic rescue in NAXDko cells. HAP1 control and NAXDko cells were grown in galactose without or with supplementation of NAD precursors (2 mM each) for 72 h or 96 h and viable cell count was measured. B) NADHX burden (calculated as the S-NADHX level divided by the NADH level) showed a decrease with either NR or NR plus inosine supplementations. The cells were grown as indicated in panel A) for 72 h in galactose without or with the indicated supplements at a concentration of 2 mM. In this preliminary experiment, R-NADHX levels could not be reliably calculated owing to co-elution with interfering compounds and therefore, the NADHX burden was calculated considering only S-NADHX levels. C) HAP1 control and NAXDko cells were grown in glucose or galactose without or with the indicated supplements (0.5 mM NR and/or 5 mM inosine) for 72 h and stained with MitoSOX Red. The images were captured at an excitation wavelength of 488 nm. Fluorescence intensity was measured as relative fluorescent units (RFUs) and normalized to control cells grown in glucose. D) Co-supplementation of inosine and nicotinamide riboside does not lead to additive effects in NAXDko cells. Control

and NAXDko cells were grown without or with the indicated supplements (5 mM each) for 72 h before measurement of the viable cell count. Absolute values are presented in Figure 7B, whereas a replot of relative cell viability values is shown here. All values are means ± SDs of at least six biological replicates (except for the NADHX burden experiment shown in panel B, where three biological replicates were analyzed and for the MitoSox experiment shown in panel C, where means ± SEMs were plotted). Statistical significance was calculated using an equal variance, unpaired Student's t-test or, for the MitoSOX measurements, an ordinary one-way ANOVA (**$p < 0.01$; ***$p < 0.001$; ****$p < 0.0001$; ns, not significant). Gal, galactose; NR, nicotinamide riboside; NA, nicotinic acid; NAM, nicotinamide mononucleotide; Ino, inosine.

Supplementary Figure S15: Preliminary characterization of iPSC-derived whole brain organoids. A) EBs derived from control and NAXDko iPSCs were compared for their size (day 4, 6 and 8 of derivation). B) The spent media from control and NAXDko WBOs were collected at the indicated time points and analyzed for absolute concentrations of glucose, glutamine, and lactate. C) Control and NAXDko WBO samples were collected at the indicated time points and immunostaining was performed using the indicated marker antibodies. For (A), measurements were from at least 24 embryoid bodies for each genotype (each dot represents one EB and the grey lines represent the median) and for (B), all values are means ± SDs of three biological replicates. For (C), representative images for each marker are shown.
